# Adapter CAR T cells to counteract T-cell exhaustion and enable flexible targeting in AML

**DOI:** 10.1038/s41375-023-01905-0

**Published:** 2023-04-27

**Authors:** D. Nixdorf, M. Sponheimer, D. Berghammer, F. Engert, U. Bader, N. Philipp, M. Kazerani, T. Straub, L. Rohrbacher, L. Wange, S. Dapa, D. Atar, C. M. Seitz, K. Brandstetter, A. Linder, M. von Bergwelt, H. Leonhardt, J. Mittelstaet, A. Kaiser, V. Bücklein, M. Subklewe

**Affiliations:** 1 Department of Medicine III, University Hospital, LMU, Munich, Germany; 2 Laboratory for Translational Cancer Immunology, LMU Gene Center, Munich, Germany; 3 Miltenyi Biotec B.V. & Co. KG, Bergisch Gladbach, Germany; 4 Core Facility Bioinformatics, Biomedical Center, LMU, Munich, Germany; 5 Anthropology and Human Genomics, Faculty of Biology, LMU, Munich, Germany; 6 Department of General Pediatrics, Hematology and Oncology, University Children’s Hospital Tuebingen, Tuebingen, Germany; 7 Cluster of Excellence iFIT (EXC 2180) “Image-Guided and Functionally Instructed Tumor Therapies”, Eberhard Karls University Tuebingen, Tuebingen, Germany; 8 Department of Biology II, LMU, Munich, Germany; 9 Gene Center and Department of Biochemistry, LMU, Munich, Germany; 10 Department of Medicine II, University Hospital, LMU, Munich, Germany; 11 German Cancer Consortium (DKTK) and German Cancer Research Center (DKFZ), Heidelberg, Germany

**Keywords:** Targeted therapies, Immunotherapy

## Abstract

Although the landscape for treating acute myeloid leukemia (AML) patients has changed substantially in recent years, the majority of patients will eventually relapse and succumb to their disease. Allogeneic stem cell transplantation provides the best anti-AML treatment strategy, but is only suitable in a minority of patients. In contrast to B-cell neoplasias, chimeric antigen receptor (CAR) T-cell therapy in AML has encountered challenges in target antigen heterogeneity, safety, and T-cell dysfunction. We established a Fab-based adapter CAR (AdCAR) T-cell platform with flexibility of targeting and control of AdCAR T-cell activation. Utilizing AML cell lines and a long-term culture assay for primary AML cells, we were able to demonstrate AML-specific cytotoxicity using anti-CD33, anti-CD123, and anti-CLL1 adapter molecules in vitro and in vivo. Notably, we show for the first time the feasibility of sequential application of adapter molecules of different specificity in primary AML co-cultures. Importantly, using the AML platform, we were able to demonstrate that chronic T-cell stimulation and exhaustion can be counteracted through introduction of treatment-free intervals. As T-cell exhaustion and target antigen heterogeneity are well-known causes of resistance, the AdCAR platform might offer effective strategies to ameliorate these limitations.

## Introduction

CAR T-cell therapies have revolutionized the treatment of various B-cell malignancies [1–3]. Although allogeneic stem cell transplantation first demonstrated the power of adoptive T-cell transfer for combating leukemia, in AML, CAR T-cell therapy is only slowly progressing. The main challenges to the successful use of CAR T cells in the AML setting are inter- and intra-patient heterogeneity in the target antigen expression profile and a lack of leukemia-restricted target antigens. The latter translates into increased toxicity and safety issues [4, 5]. Due to the lack of fast and efficient safety switches to circumvent on-target-off leukemia toxicity, current CAR T constructs are mainly used as a bridge to transplant strategy [6, 7].

Another cause of CAR T-cell failure is T-cell dysfunction, the reasons for which are multifaceted, ranging from the quality of the T cells at the time of apheresis [8, 9], remodeling by the tumor microenvironment [10–14], chronic antigen exposure [15, 16], also caused by the intrinsic resistance of AML cells due to impaired death receptor signaling [17], and tonic CAR T-cell signaling [18, 19].

Building on the previously described Adapter CAR^TM^ T-cell platform [20] we therefore developed a fragment antigen-binding region (Fab)-based approach that enables flexibility of targeting and control of AdCAR T-cell activation. Using an AdCAR directed against a biotinylated adapter molecule (AM) in the context of a specific linker, we demonstrate that AdCAR T cells are highly functional against multiple AML cell lines in vitro and in vivo, and against primary AML (pAML) cells, by utilizing a long-term culture system in combination with αCD33, αCD123, and αCLL-1 AMs.

The majority of AML-associated target antigens get internalized, hence we also studied receptor-mediated endocytosis of various AM formats and its impact on half-life and cytotoxicity. In addition, we provide the first evidence that serial use of AMs against different target antigens is feasible and highly potent in eliminating pAML cells in ex vivo long-term co-cultures. To counteract CAR T-cell exhaustion as a result of continuous (CONT) stimulation, we sought to advantageously utilize the AM as an on–off switch for implementing treatment free intervals (TFIs). Importantly, we could show that the AdCAR T-cell platform allows the use of TFIs that abrogate target-induced T-cell dysfunction.

## Methods

### In vitro cytotoxicity assays and pAML culture

AdCAR T cells or untransduced (mock) T cells were co-cultured with MV4-11, HL-60, or OCI-AML-3 cells at varying E:T ratios in the presence of either αCD33, αCD123, αCLL-1, or αCD19 AMs. Specific lysis was assessed by flow-cytometry on Cytoflex S/LX instruments (Beckman Coulter, Brea, CA, US) at the iFlow Core facility, Munich, and calculated relative to conditions without AMs or mock T cells. pAML long-term co-culture assays were performed as described [21]. For serial targeting experiments, AMs were either replenished or exchanged for an AM with different target specificity every 3 days by exchanging 50% of the cell culture medium with 2x complete blast medium containing the AM.

### Internalization assays

αCD33-AM_Fab_ was labeled with pHrodo Red Avidin (ThermoFisher Scientific, Waltham, MA) according to manufacturer’s instructions. MV4-11 cells were labeled with 500 ng/ml αCD33-AM_Fab_-pHrodo and 1:1000 Hoechst 33342 (ThermoFisher Scientific) for 15 min at 4 °C. Unbound AM was removed and cells were incubated for 6 h at 4 °C or 37 °C on poly-D-lysine-coated glass-bottomed two-well ibidi slides (ibidi GmbH, Gräfelfing, Germany). Images were acquired on a Nikon TiE microscope. Instrument settings are outlined in detail in the supplements.

#### Indirect internalization assay

AML cells were treated for 15 min at 4 °C with an AM, after which time unbound AM was removed. Cells were incubated for 0–6 h at 4 °C or 37 °C in R10. At each time point, cells were washed and stained with anti-biotin-PE (Miltenyi Biotec, Bergisch Gladbach, Germany) and AquaLive/Dead (Invitrogen) for 15 min at 4 °C. T cells were analyzed by flow-cytometry. The percentage of surface-bound AMs was calculated based on the MFI at 37 °C compared to controls.

### In vitro long-term AdCAR T-cell stimulation and treatment-free intervals

Healthy donor (HD)-derived AdCAR T cells were co-cultured with irradiated (2.5 Gy) OCI-AML-3 cells in R10 (E:T = 1:4; 1 × 10^6^ cells/ml) in the presence of 10 ng/ml αCD33-AM_Fab_. After 3 days, half of the medium was exchanged with fresh medium containing αCD33-AM_Fab_ and irradiated target cells (E:T = 1:2). On day 7, human T cells were isolated using the EasySep Human CD3 Positive Selection Kit II (Stemcell, Vancouver, Canada) according to manufacturer’s instructions. A fraction of isolated T cells was used for functional cytotoxicity and proliferation assays and immunophenotyping [22]. The remaining T cells were re-cultured with irradiated target cells (E:T = 1:4). To implement TFIs, the cultures were split and treated with or without αCD33-AM_Fab_ for a further 7 days. A third CONT round of αCD33-AM_Fab_ stimulation was performed until day 21. Co-culture supernatants were harvested to quantify human cytokine secretion.

### In vivo studies

All experiments were performed according to the guidelines of the Federation of European Laboratory Animal Science Associations (FELASA) in the animal husbandry facilities of Miltenyi Biotec. General health status was monitored daily. NOD.Cg-*Prkdc*^*scid*^ *Il2rg*^*tm1Wjl*^/SzJ mice were engrafted with OCI-AML-2 cells via tail vein injection on day −5. AdCAR T or conventional CD33CAR T cells were injected intravenously on day 0. αCD33-AM_Fab_ was intraperitoneally administered daily. Leukemia growth was monitored twice per week by bioluminescence imaging (BLI). A detailed description of the in vivo studies is provided in the supplements.

## Results

### AdCAR T cells mediate specific lysis of AML cell lines

**Fig. 1:**
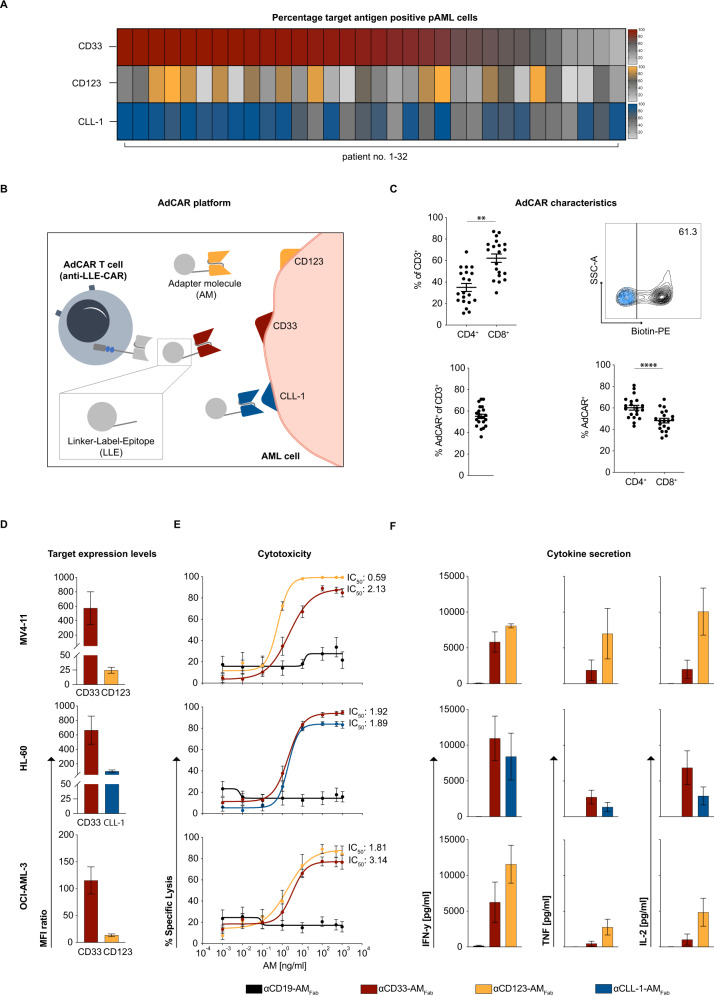
AdCAR T cells mediate specific lysis of AML cell lines. **A** Percentage of CD33-, CD123- and CLL-1-positive pAML cells assessed by surface marker staining with biotinylated AMs and subsequent secondary staining (*n* = 32). **B** Schematic illustration of the AdCAR T-cell platform recognizing an AML cell via AMs directed against the target antigens CD33, CD123, or CLL-1. **C** AdCAR characteristics after transduction and 14 days of expansion in IL-7/IL-15. The CD4/CD8 ratio of the AdCAR T-cell product was determined by flow-cytometry (*n* = 20). The transduction efficiency of AdCAR T cells was measured by biotin-PE staining. A representative contour plot depicts the percentage of AdCAR^+^ fraction in black (untransduced cells in blue). **D** Target antigen expression on AML cell lines determined by surface marker staining with biotinylated AMs and subsequent secondary staining (*n* = 3). MFI ratios were calculated based on corresponding controls without AMs. **E** AdCAR T-cell-mediated cytotoxicity after 48 h (*n* = 4–12) against the AML cell lines MV4-11, HL-60, and OCI-AML-3 (E:T = 1:1) in co-cultures containing αCD33-AM_Fab_, αCD123-AM_Fab_, or αCLL-1-AM_Fab_ at concentrations ranging from 1 pg/ml to 1000 ng/ml. Target-irrelevant αCD19-AM_Fab_ was used as a control AM. Specific lysis was calculated relative to the mock T-cell condition. **F** Secretion of IFN-γ, TNF, and IL-2, determined by cytometric bead array (CBA) analysis, from corresponding cytotoxicity assays at an AM concentration of 10 ng/ml (*n* = 3). All graphs present the mean ± SEM. Statistical analysis: paired *t*-test; ns *p* > 0.05; *p < 0.05; **p < 0.01; ***p < 0.001; ****p < 0.0001.

One major obstacle to CAR T-cell-based immunotherapy in AML is the heterogenous expression profile of target antigens. We analyzed 32 pAML samples at initial-diagnosis for their expression of the target antigens CD33, CD123, and CLL-1. The majority expressed these antigens at high levels, however, we did observe samples in which a minority of cells expressed one of the three antigens (Fig. 1A, Supplementary Fig. S1A and Table 1). Hence, to make CAR T-cell therapy applicable in all AML subtypes and at the same time counteract antigen escape variants, a CAR T platform allowing to address several target antigens either in parallel or sequentially is desirable. We therefore established an AdCAR T-cell platform allowing flexible targeting of various antigens by uncoupling antigen recognition and T-cell activation (Fig. 1B).

**Table 1:** Patient characteristics.

Patient no.	Age	ELN	FAB	% positive			MFI ratio		
-	-	-	-	CD33	CD123	CLL1	CD33	CD123	CLL1
1	68	adverse	N/A	99.9	43.8	99.8	104.1	2.6	55.9
2	69	adverse	N/A	99.6	51.5	99.5	204.7	4.6	144.9
3	N/A	N/A	N/A	99.5	90	96.3	105.3	19.4	78.5
4	28	adverse	N/A	99.4	99.4	82.8	418.4	68.9	43.2
5	N/A	N/A	N/A	99.2	85.4	99	84.9	8.3	79.8
6	39	N/A	N/A	99	3.99	87.7	41.7	4.3	25.7
7	N/A	N/A	N/A	98.9	82.6	90.8	20.1	3.2	14.3
8	71	adverse	M5	98.7	5.43	98.2	237.7	1.9	198.4
9	79	adverse	M1	98.1	73.2	99	116.7	26.6	377.7
10	N/A	N/A	N/A	98.1	33.1	88.3	41.7	4.3	25.7
11	N/A	N/A	N/A	98.1	78.1	97	141.4	28.9	94.2
12	N/A	N/A	N/A	97.5	34.8	68.3	39.8	6.1	12.6
13	77	adverse	M1	95.7	91.7	47.1	35.9	26.6	6.2
14	N/A	N/A	N/A	95.7	21.7	95.4	187.3	9.4	152.8
15	N/A	N/A	N/A	94.8	3.81	48.8	18.8	1.3	3.9
16	70	adverse	M1	92.5	63.4	66.5	74.7	25.7	34.1
17	N/A	N/A	N/A	91.6	43.9	76.6	11.7	4.1	8.5
18	81	adverse	M2	87.6	25.6	37.6	14.0	3.5	4.6
19	N/A	N/A	N/A	86.6	34.1	94.5	73.9	6.2	125.3
20	49	adverse	M4	83.5	81	55.5	37.5	29.4	17.9
21	N/A	N/A	N/A	79.2	99.1	100	50.4	172.0	1363.9
22	57	adverse	N/A	69	33	48.4	73.7	22.5	41.6
23	34	adverse	M0	67.9	24.7	44.4	65.1	6.1	14.2
24	N/A	adverse	N/A	65.8	75.1	78.2	25.1	26.8	81.2
25	74	intermediate	N/A	63.5	61.6	72.5	22.5	26.4	45.2
26	83	adverse	N/A	62.9	11.6	81.6	22.3	2.0	157.7
27	63	adverse	M0	58.4	97.1	52.6	16.2	56.4	17.1
28	N/A	adverse	N/A	50.5	51.1	48.8	22.0	27.6	24.1
29	66	adverse	M5a	31.7	1.93	40	21.7	4.0	40.7
30	N/A	N/A	N/A	30.5	7.67	89.6	6.0	2.4	43.9
31	N/A	N/A	N/A	28.6	53.9	67.2	4.4	5.6	13.7
32	N/A	N/A	N/A	18.5	29	95.9	62.1	1.5	100.7

Lentiviral transduction of human T cells with the AdCAR construct was consistent and highly efficient (% transduction efficiency: 55 ± SEM), whereas CD4^+^ T cells were significantly more susceptible to lentiviral transduction than CD8^+^ T cells (Fig. 1C and Supplementary Fig. S1B).

HD-AdCAR T cells were co-cultured with different AML cell lines (MV4-11, HL-60, OCI-AML- 3) expressing CD33, CD123, and CLL-1 at various levels (Fig. 1D) in the presence of target-antigen-specific Fab- or Ab-based AMs. Specific cytotoxicity was observed against all three AML cell lines and was dependent on target-antigen specificity, antigen density, AM concentration, and E:T ratio (Fig. 1E and Supplementary Fig. S1C–F).

Addition of the control αCD19-AM_Fab_ did not result in nonspecific lysis (Fig. 1E), and we found no target-antigen-independent cytotoxicity for the AMs (Supplementary Fig. S1E). AdCAR T-cell activation was measured by the secretion of the effector cytokines IFN-γ, TNF, and IL-2, and was observed only in the presence of target-antigen-specific AMs and corresponding target cells (Fig. 1F).

### AdCAR-mediated cytotoxicity against pAML cells: impact of receptor-mediated internalization on AdCAR T-cell efficacy

**Fig. 2:**
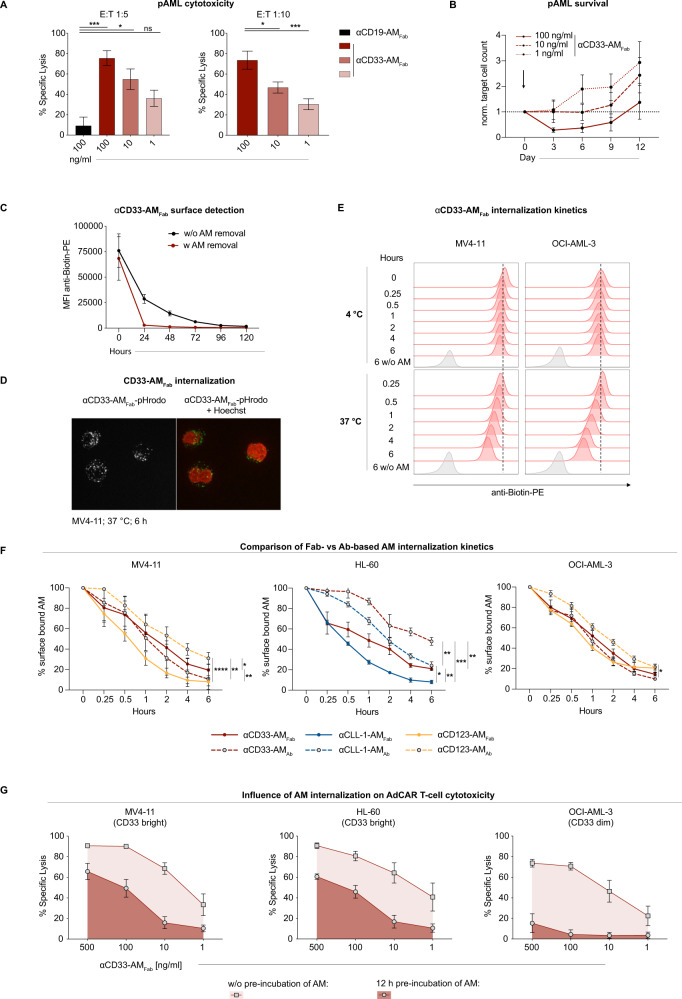
AdCAR-mediated cytotoxicity against pAML cells: impact of receptor-mediated internalization on AdCAR T-cell efficacy. **A** AdCAR T-cell-mediated cytotoxicity after 72 h (*n* = 3–13) against pAML cells co-cultured on irradiated MS5 feeder cells (E:T = 1:5 and 1:10) in the presence of 100, 10 or 1 ng/ml αCD33-AM_Fab_. αCD19-AM_Fab_ at 100 ng/ml served as a negative control. Specific lysis was calculated relative to the AdCAR T-cell condition without AM. **B** Growth of pAML samples in long-term co-cultures with AdCAR T cells (E:T = 1:10) for 12 days with initial (one-time) addition of 100, 10 or 1 ng/ml αCD33-AM_Fab_. pAML cell counts over time are plotted as normalized target cell count relative to starting conditions on day 0 (*n* = 4–9). **C** Levels of receptor-bound αCD33-AM_Fab_ were monitored daily for 5 days on MV4-11 cells stained with 500 ng/ml AM. AM was added on day 0 for 15 min at 4 °C, unbound AM was either removed from the supernatant or not (*n* = 3–4). **D** Internalization assay. Left: Representative confocal image of MV4-11 cells stained for 6 h at 37 °C with 500 ng/ml αCD33-AM_Fab_ coupled to pHrodo Red Avidin (gray). Right: Nuclei were stained with Hoechst 33342 (red) and merged with the pHrodo Red Avidin (green) channel. AM internalization can be seen as puncta located in the cytoplasm. Control conditions at 4 °C did not yield a measurable pHrodo Red Avidin signal (data not shown). Three independent experiments were performed. **E** Representative example of flow-cytometry-based indirect internalization assay of αCD33-AM_Fab_ at 4 °C and 37 °C for 6 h on MV4-11 and OCI-AML-3 cells. AML cells were labeled for 15 min at 4 °C with 500 ng/ml AM. The unbound AM was removed and the percentage of surface-bound AM (red histograms) was assessed at each indicated time point by secondary staining with anti-Biotin-PE antibody. **F** Quantitative representation of the internalization assay described in **E** (*n* = 6). The kinetics of internalization of Fab- and Ab-based AMs were compared on AML cell lines. **G** The influence of AM internalization on AdCAR T-cell-mediated cytotoxicity (*n* = 6). AML cell lines were pre-incubated for either 0 or 12 h with 500–1 ng/ml αCD33-AM_Fab_ before addition of AdCAR T cells (E:T = 1:1). Cytotoxicity was assessed by flow-cytometry after an additional 48 h. Data are plotted as mean ± SEM. Statistical analysis: **A** Ordinary one-way ANOVA with Dunnett’s comparison; **F** Mixed-effects analysis with Geisser–Greenhouse correction. ns *p* > 0.05; *p < 0.05; **p < 0.01; ***p < 0.001; ****p < 0.0001.

Next, we assessed whether AdCAR T cells can effectively target and lyse pAML cells. We focused on CD33 as the target antigen and observed high and specific lysis of pAML cells (% specific lysis: 75 ± SEM at 100 ng/ml αCD33-AM_Fab_) after 3 days of co-culture with AdCAR T cells using our previously described pAML culture system [21]. Target elimination was AM dose and E:T ratio dependent (Fig. 2A). Notably, AdCAR T cells generated from HD or pAML T cells were equally effective in vitro (Supplementary Fig. S2C), suggesting the vector system is suitable for clinical applications.

However, over an extended period of 12 days, we observed an outgrowth of pAML cells with faster kinetics at lower AM concentrations (Fig. 2B). In line with this, T-cell proliferation peaked at around day 6 (Supplementary Fig. S2A) and the percentage of PD1/TIM3/LAG-3^+^ T cells declined over time, indicating a loss of T-cell activation (Supplementary Fig. S2B).

Because the AMs were added only at the start of the co-cultures, we speculated that the available AMs were consumed by target antigen receptor-mediated endocytosis. We assessed the surface retention of αCD33-AM_Fab_ bound to the CD33 receptors of MV4-11 AML cells, by labeling the cells with AMs in the absence of AdCAR T cells. We observed almost complete loss of receptor-bound AMs within 72 h, with even faster kinetics after prior removal of unbound AM, suggesting that free AM was binding to recycled or newly synthesized antigen receptors (Fig. 2C).

To further validate our findings, αCD33-AM_Fab_ was coupled to a pH-sensitive dye. Confocal microscopy confirmed that internalization of AMs occurred rapidly at 37 °C (Fig. 2D). Furthermore, a deficiency of CD33 receptors, as well as blocking vesicle transport with monensin, completely disrupted internalization by MV4-11 cells, hinting convincingly at receptor-mediated endocytosis of the AMs (Supplementary Fig. S2D).

To quantitatively describe this phenomenon, we characterized the internalization kinetics of different AM formats (Fab vs Ab based). We observed a rapid decline of all analyzed AMs within the first 2 h (Fig. 2E, F and Table 2), with kinetics dependent on the AML cell line, AM format, and the target antigen. Interestingly, Fab-based AMs had shorter half-lives than the Ab-based formats, and, after 6 h, only 19%, 8%, and 8% of the initial surface-bound concentrations of αC33-AM_Fab_, αCD123 AM_Fab_ (both on MV4-11), and αCLL-1-AM_Fab_ (on HL-60), respectively, were detected. To validate that AM internalization also occurs in a clinically relevant setup, we confirmed the results with pAML cells (Supplementary Fig. S2E). Of note, based on the detection of surface-bound AM we cannot exclude AM-dissociation and potentially other elimination pathways, having contributed to the decrease of AM.

**Table 2:** Adapter molecule internalization kinetics.

half-lives [min]	MV4-11	HL-60	OCI-AML-3
aCD33-AM_Fab_	86	127	73
aCD33-AM_Ab_	64	88	65
aCD123-AM_Fab_	36	N/A	43
aCD123-AM_Ab_	56	N/A	71
aCLL-1-AM_Fab_	N/A	18	N/A
aCLL-1-AM_Ab_	N/A	86	N/A

To gain a deeper understanding into whether and how AM internalization influences AdCAR T-cell cytotoxicity, we performed co-culture cytotoxicity assays with three AML cell lines expressing high or low target antigen levels. AML cells were labeled with αCD33-AM_Fab_ and AdCAR T cells were added 12 h later. Consistent with the internalization study, cytotoxicity was also dependent on AM level, as lysis of target cells declined upon prolonged pre-incubation of AML cells with AMs (Fig. 2G). Importantly, this effect was most pronounced if target antigen densities were low (OCI-AML-3 cell line), leading to almost complete loss of AdCAR T-cell-mediated cytotoxicity, even at initial αCD33-AM_Fab_ concentrations of 500 ng/ml (decrease of specific lysis: 73% to 15% ± SEM). We did not observe long-term downmodulation of CD33 expression levels by AM internalization (Supplementary Fig. S2F).

In summary, we showed that AdCAR T cells efficiently lyse pAML cells. However, we describe AM internalization as a common phenomenon for a variety of Fab- and Ab-based AM formats that target known internalizing antigens. AM internalization reduced their respective serum levels, thereby contributing to a form of “antigen sink” that impairs AdCAR T-cell cytotoxicity, especially at low levels of target antigen.

### AdCAR T cells allow for sequential targeting of pAML cells

Based on the short half-lives of the AMs, we examined whether repetitive AM dosing prolongs AdCAR T-cell functionality and leads to better elimination of pAML cells compared to single administration. Owing to the heterogeneity of target antigen expression in AML, we took advantage of the versatility of the AdCAR technology, which enables a sequential application of AMs against different target antigens. AMs against CD33, CD123, or CLL-1 were used once at a concentration of 10 ng/ml or replenished every third day until day 12 of co-cultures of pAML cells and AdCAR T cells (E:T = 1:10). Additional experiments included a dose increase to 100 ng/ml and/or a switch to an AM of different target specificity on day 6 of co-culture (10 or 100 ng/ml), followed by AM replenishment on day 9.

**Fig. 3:**
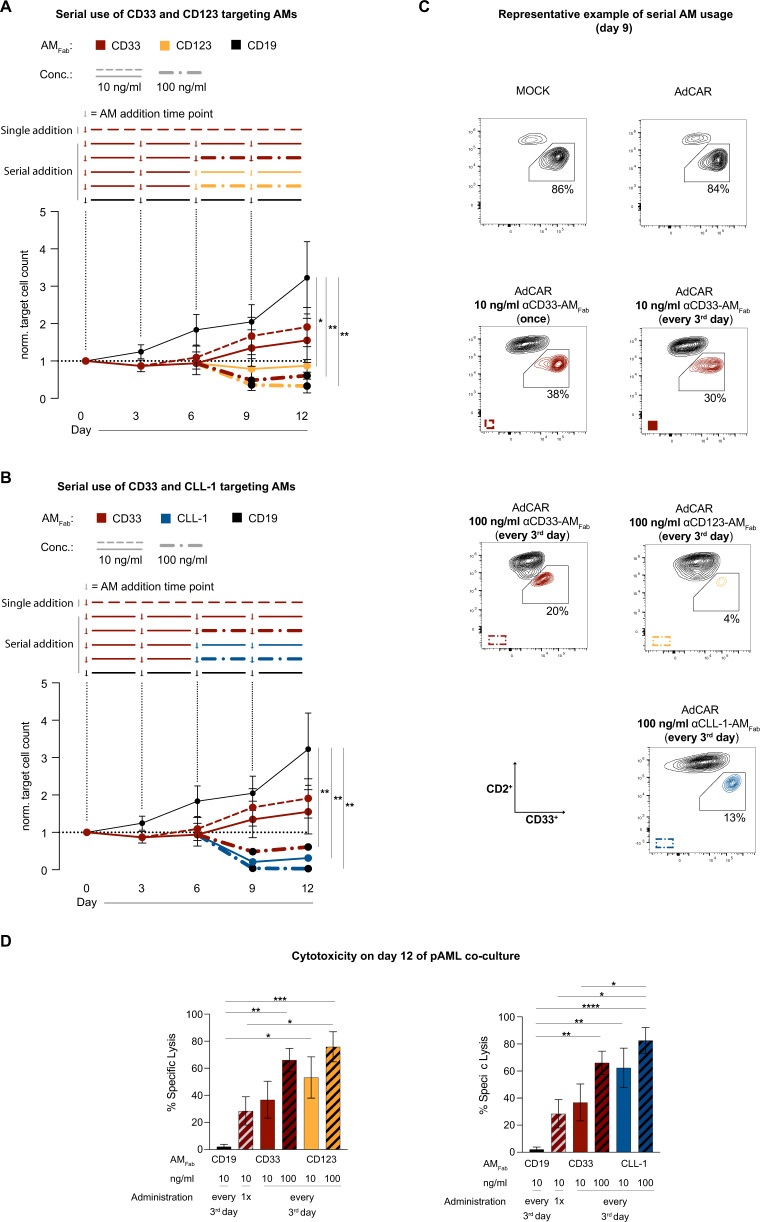
AdCAR T cells allow for sequential targeting of pAML cells. **A**, **B** Long-term (12 days) co-cultures of AdCAR T cells and pAML cells (E:T = 1:10). pAML cell counts over time are plotted as normalized target cell count relative to starting conditions on day 0 (*n* = 7). αCD19-AM_Fab_ was replenished every third day at 10 ng/ml and served as a control. αCD33-AM_Fab_ was either applied once (10 ng/ml; dotted red line) or every third day until day 6 (solid red line). On days 6 and 9, the AM dose was either maintained at 10 ng/ml or increased to 100 ng/ml (bold dotted line). Alternatively, AMs were switched to AMs of different target specificity (αCD123-AM_Fab_ or αCLL-1-AM_Fab_) on days 6 and 9 (10 or 100 ng/ml). **C** Representative flow-cytometry data from day 9 of co-culture. T cells and pAML cells were distinguished by CD2 and CD33 staining, respectively. Doublets, as well as dead cells were excluded, as described. **D** Corresponding AdCAR T-cell-mediated cytotoxicity on day 12 of co-culture. Specific lysis was calculated relative to the AdCAR T-cell condition without AM. Data are presented as mean ± SEM. Statistical analysis: Ordinary one-way ANOVA with Dunnett’s comparison; ns *p* > 0.05; *p < 0.05; **p < 0.01; ***p < 0.001; ****p < 0.0001.

All AMs reduced leukemia growth compared to αCD19-AM_Fab_ controls, underlining the specificity and potency of the AdCAR platform. However, a single addition of 10 ng/ml of αCD33-AM_Fab_ or αCLL-1-AM_Fab_ was insufficient to stop leukemia growth (Fig. 3A, B and Supplementary Fig. S3C, D).

Repetitive administration of AMs at 10 ng/ml further delayed (αCD33-AM_Fab_) or halted (αCD123-AM_Fab_ or αCLL-1-AM_Fab_) leukemia outgrowth compared to a single addition of the AM.

Convincingly, an increase in dose of αCD33-AM_Fab_ or αCLL-1-AM_Fab_ from 10 to 100 ng/ml on day 6 of co-culture resulted in almost complete elimination of AML blasts (Fig. 3A, B), highlighting the potential for individually adjusting treatment conditions based on response to therapy or on target antigen levels.

Interestingly, switching from αCD33-AM_Fab_ on day 6 to 10 ng/ml of either αCD123-AM_Fab_ or αCLL-1-AM_Fab_ was effective, demonstrating comparable efficacy in eliminating pAML cells to 100 ng/ml αCD33-AM_Fab_. AdCAR T-cell-mediated cytotoxicity was further enhanced not only by changing the target specificity but also by increasing the respective AM doses to 100 ng/ml (Fig. 3A–D). Notably, targeting the same pAML samples first with αCLL-1-AM_Fab_ followed by αCD33-AM_Fab_ or αCD123-AM_Fab_ did not result in higher lysis compared to continuous αCLL-1-AM_Fab_ targeting, as opposed to starting the treatment with αCD33-AM_Fab_ (Supplementary Fig. S3C–E).

The effects on AdCAR T-cell cytotoxicity were accompanied by a trend for increased T-cell proliferation after AM switching or dose increases (day 9), as well as increased expression of activation markers, indicative of pronounced and sustained AdCAR T-cell activation (Supplementary Fig. S3A, B, F, G).

These results collectively show that the use of highly modular AdCAR technology potently and specifically eradicated pAML cells in a dose- and time-dependent manner.

### Fab molecules efficiently activate AdCAR T cells in vivo

**Fig. 4:**
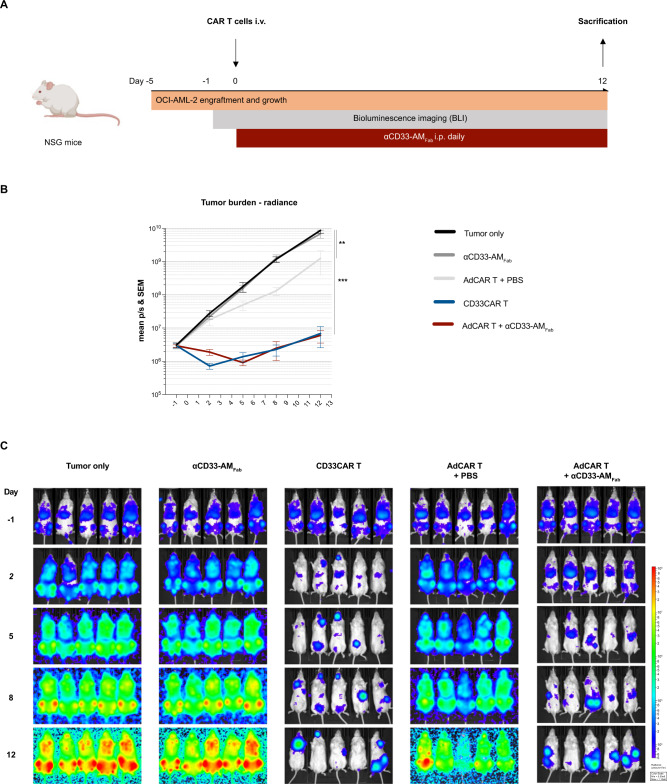
Fab molecules efficiently activate AdCAR T cells in vivo. **A** Schematic representation of the in vivo experimental timeline: NSG mice were inoculated on day −5 with luciferase-expressing OCI-AML-2 tumor cells followed by injection of AdCAR/CAR T cells on day 0. A second-generation conventional CD33CAR T-cell construct served as control. AdCAR/CAR T-cell functionality was assessed regularly by BLI of OCI-AML-2 cells. **B** In vivo BLI of OCI-AML-2 cells. **C** Bioluminescence images (*n* = 5 mice per group). Data are plotted as mean ± SEM. Statistical analysis: Ordinary one-way ANOVA with Tukey’s comparison; ns *p* > 0.05; *p < 0.05; **p < 0.01; ***p < 0.001; ****p < 0.0001.

Next, we aimed to translate our findings to a clinically relevant AML in vivo model by testing whether Fab-based AMs were able to efficiently direct AdCAR T cells against a highly aggressive OCI-AML-2 model. Therefore, HD-AdCAR T cells were expanded in vitro for 8 days and transferred to NSG mice bearing OCI-AML-2 leukemias (Fig. 4A). The mice were injected daily with αCD33-AM_Fab_. AdCAR T cells were readily activated in vivo using Fab-based AMs. Convincingly, AdCAR T cells showed equipotency in controlling leukemia growth compared to conventional CD33CAR T cells, as quantified by BLI (Fig. 4B, C).

### Treatment-free intervals prolonged AdCAR T-cell function

**Fig. 5:**
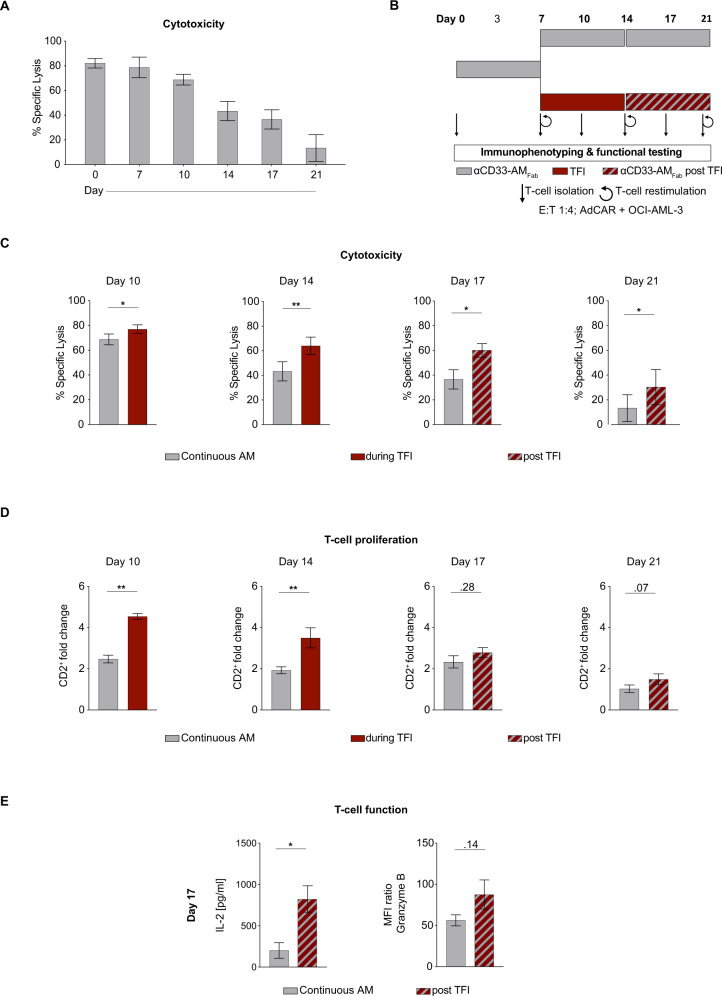
Treatment-free intervals prolong AdCAR T-cell function in vitro. **A** AdCAR T cells were continuously stimulated for 21 days with 10 ng/ml αCD33-AM_Fab_ in the presence of irradiated OCI-AML-3 cells (E:T = 1:4; *n* = 3–12). OCI-AML-3 cells and AMs were replenished every third day. AdCAR T cells were isolated at the indicated days and cytotoxicity against OCI-AML-3 cells (E:T = 1:1) after 72 h was assessed by flow-cytometry. **B** Timeline and overview of the continuous and intermittent stimulation of AdCAR T cells co-cultured with OCI-AML-3 cells over 21 days. **C** Cytotoxicity of AdCAR T cells isolated from co-cultures against OCI-AML-3 cells at the indicated days (*n* = 3–12; E:T = 1:1; 10 ng/ml αCD33-AM_Fab_). **D** T-cell proliferation expressed as fold change in CD2^+^ cells compared to conditions without AM (*n* = 5–12). **E** IL-2 secretion determined by CBA analysis of co-culture supernatants on day 17 (*n* = 6) and granzyme B expression of CD8^+^ AdCAR T cells isolated on day 17 and transferred to 72 h cytotoxicity assays (*n* = 5). Data are presented as mean ± SEM. Statistical analysis: paired *t*-test; ns *p* > 0.05; *p < 0.05; **p < 0.01; ***p < 0.001; ****p < 0.0001.

T-cell exhaustion is an emerging cause of CAR T-cell failure. We previously developed an in vitro system to monitor T-cell dysfunction induced by continuous bispecific antibody (BsAb) exposure in a clinically relevant B-cell lymphoma model [22]. It is unknown if AdCAR T cells react to CONT stimulation in the same way as conventional CAR T cells. Here, we adapted the long-term stimulation system to further evaluate AdCAR T-cell exhaustion in the context of AML. AdCAR T cells were co-cultured for 21 days with OCI-AML-3 in the presence of αCD33-AM_Fab_. AdCAR T cells were isolated on days 0, 7, 10, 14, 17, and 21, and their cytotoxicity against OCI-AML-3 cells was assayed in co-cultures. We observed progressive AdCAR T-cell dysfunction over time, starting between day 10/14 of co-culture (Fig. 5A; 82% specific lysis on day 0 vs 13% on day 21), supporting our hypothesis that prolonged periods of AdCAR T-cell activation lead to loss of effector function.

As previously shown by us and others, intermittent T-cell stimulation using TFIs or molecular and chemical switches can prolong T-/CAR T-cell functionality, mainly through transcriptional and epigenetic remodeling [19, 22, 23].

Hence, we compared continuously to intermittently stimulated AdCAR T cells in our AML-optimized in vitro dysfunction model for 21 days. In both conditions, AdCAR T cells were co-cultured with OCI-AML-3 target cells in the presence of αCD33-AM_Fab_ for 7 days. Then, AdCAR T cells were either exposed for another 7 days to AMs and OCI-AML-3 cells or cultured in the absence of AMs. All cultures were then treated for a further 7 days with αCD33-AM_Fab_ (Fig. 5B). AdCAR T cells were again isolated on days 0, 7, 10, 14, 17, and 21, and their cytotoxicity was assayed in co-cultures.

We observed significantly improved AdCAR T-cell-mediated cytotoxicity against OCI-AML-3 cells during and after the TFI (specific lysis ± SEM: day 14, CONT vs TFI = 43% vs 64%; day 17, CONT vs post TFI = 36% vs 60%; Fig. 5C). In addition, AdCAR T cells intermittently exposed to AMs demonstrated greater proliferation, IL-2 secretion, granzyme B production, and increased expression of PD1 and LAG-3 (Fig. 5D, E and Supplementary Fig. S4A). The AdCAR receptor was not differentially expressed between the two treatment modes (Supplementary Fig. S4B). Whereas T-cell subset analysis revealed a shift towards an effector memory subtype during CONT AM stimulation (Supplementary Fig. S4C), the resting period had no effect on this, indicating that the functional improvement is not driven by only a subpopulation of AdCAR T cells.

In summary, we showed that continuous AdCAR T-cell stimulation leads to a decrease in effector functions, which can be abrogated by the use of TFIs.

### Treatment-free intervals lead to transcriptional reprograming of AdCAR T cells

**Fig. 6:**
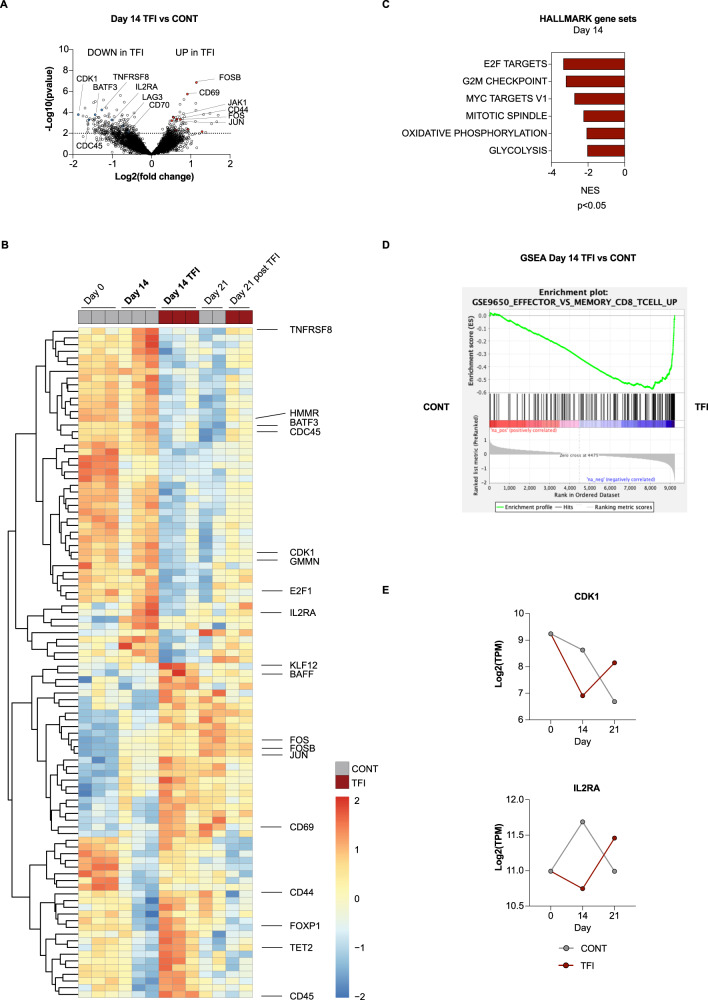
Treatment-free intervals lead to transcriptional reprograming of AdCAR T cells. **A** Volcano plot of DEGs in day 14 TFI-treated versus CONT-treated AdCAR T cells; *p* < 0.01. Selected genes are highlighted in blue (downregulated) or red (upregulated). **B** Heatmap with hierarchical clustering of the top 100 DEGs in day 14 TFI-treated versus CONT-treated AdCAR T cells; *p* < 0.01. Selected genes are indicated. **C** Hallmark gene set analysis of day 14 TFI-treated versus CONT-treated AdCAR T cells; *p* < 0.05. **D** GSEA of day 14 TFI-treated versus CONT-treated AdCAR T cells using MSigDB and the gene set GSE9650_EFFECTOR_VS_MEMORY_CD8_TCELL_UP [15]. **E** Log_2_(TPM) values of CDK1 and IL2RA over time for TFI-treated and CONT-treated AdCAR T cells. DEG = differentially expressed gene; NES = normalized enrichment score; GSEA = gene set enrichment analysis.

To better understand how functional superiority is established, we performed bulk RNA sequencing on isolated AdCAR T cells from CONT- and TFI-treated cells at days 0, 14, and 21 from co-cultures from three individual donors. An analysis of differentially expressed genes (DEGs) of day 14 AdCAR T cells identified 115 significantly upregulated and 219 downregulated genes under TFI conditions versus CONT (*p* < 0.01; Fig. 6A). Unsupervised clustering showed markedly different gene expression patterns under the two treatment modes, indicating transcriptional reprograming. Most importantly, genes related to activation (IL2RA, CD70, LAG3) and cell cycle (CDK1, GMMN, E2F1, CDC45) were downregulated in day 14 TFI-treated AdCAR T cells compared to CONT-stimulated cells, consistent with functional rest (Fig. 6A, B). Interestingly, these genes remained downregulated in day 21 CONT-stimulated AdCAR T cells, indicating a progressive loss of cellular activity due to sustained antigen stimulation. In contrast, other genes related to T-cell activation (CD69, CD44, CD45, Jak1) were upregulated on day 14 TFI-treated relative to CONT-treated AdCAR T cells, pointing towards a better effector function. Pathway comparison of day 14 TFI- and CONT-treated AdCAR T cells was consistent with downregulation of cell cycle (E2F targets, normalized enrichment score, NES = − 3.35; G2M checkpoint, NES = − 3.21; MYC targets V1, NES = − 2.74; mitotic spindle, NES = − 2.25; *p* < 0.05) and metabolism-associated genes (OXPHOS, NES = − 2.08; glycolysis, NES = − 2.04; *p* < 0.05), highlighting AdCAR T-cell quiescence during TFIs (Fig. 6C). Compared to a model of chronic LCM virus infection [15], gene set enrichment analysis (GSEA) revealed a shift towards memory-related from effector-related genes in day 14 TFI-treated AdCAR T cells (Fig. 6D; GSE9650, NES = − 2.41, false-discovery rate *q* = 0.0).

These data imply that day 14 TFI-treated AdCAR T cells undergo rejuvenation through transcriptional reprograming. Convincingly, although we observed progressive downregulation of cell cycle (CDK1) or activation markers (IL2RA) in CONT-stimulated AdCAR T cells, resting periods led to a re-expression of these markers on day 21 TFI-treated cells (Fig. 6E). Overall, genes and pathways downregulated during day 14 TFI were upregulated again at day 21 (and vice versa; Supplementary Fig. S5A–C). GSEA showed that in contrast to day 14, effector-related versus memory-related genes were enriched at day 21 (Supplementary Fig. S5D). Collectively, these data suggest that day 21 TFI-treated AdCAR T cells are more functional than CONT-treated cells and have a greater potential for being re-activated upon αCD33-AM_Fab_ re-exposure. Notably, the re-expression of cell-cycle-related genes in day 21 TFI-treated AdCAR T cells did not reach the level on day 0, indicating that T-cell dysfunction cannot be completely reversed.

## Discussion

Translating the success of CD19-directed CAR T cells in B-cell neoplasms to myeloid malignancies, in particular AML, remains challenging. The lack of leukemia-restricted antigens in the myeloid compartment has hampered the advancement of T-cell-recruiting strategies, including BsAbs and CAR T cells, in AML. In addition, inter- and intra-patient heterogeneity at the genetic and protein levels renders this disease more challenging to treat using novel immunotherapeutic strategies [24]. To overcome these barriers, more individualized and safer treatment regimens are necessary.

We therefore optimized our recently developed AdCAR T-cell technology [20] for the treatment of AML. Using Fab- and Ab-based AMs against three different AML target antigens (CD33, CD123, and CLL-1), we demonstrate the specificity and efficacy of AdCAR T cells against AML cell lines and pAML samples in vitro and in vivo.

Although efficacy was already achieved in clinical trials using CAR T cells directed against CD123 or combinations of CD33/CLL-1, these approaches will only benefit a minority of patients owing to their application as a bridge to transplant for avoiding profound on-target/off-leukemia activities [6, 7]. To capitalize on the flexibility of our AdCAR T-cell platform, we demonstrated for the first time here, in an ex vivo long-term pAML model, that sequential use of AMs with different target specificity is feasible. AdCAR T cells could be readily re-targeted against CD33, CD123 or CLL-1 and activated in an AM dose-dependent manner. Notably, we cannot rule out that the observed functional benefits of eliminating pAML cells by AdCAR T cells after AM switching were impacted by the intrinsic differences of the AMs, rather than resulting from targeting antigen-escaped tumor cells.

In the context of B-cell malignancies, the mechanisms of CD19 escape variants have been well described [25]. Therefore, it appears likely that antigen escape variants will also occur in AML after single-antigen targeting, either due to pre-existing target antigen dim/neg clones or adaptive escape mechanisms. Although the sequential administration of CD19-, CD20-, or CD22-directed CAR T cells has proven efficacy [26–28], these approaches require manufacturing of multiple CAR T-cell products, emphasizing the need for flexible targeting of multiple antigens to overcome the evolution of escape variants that besets the use of a single CAR T-cell platform.

Furthermore, the majority of current mono- or dual-targeting CAR T constructs designed for AML do not integrate safety switches. Therefore, these constructs come with the inherent risk of potentially life-threatening cytokine release syndrome (CRS), ICANS and severe hematotoxicity. In fact, high-grade CRS and myeloablation have been observed in a number of pilot CAR T-cell studies targeting AML and accordingly, are commonly used as a bridge to transplant strategy [7, 29]. Hematotoxicity has also been reported utilizing CD33 or CD123 BsAb [30, 31], albeit our knowledge on long-term impact is still limited. Hence, we expect our AdCAR T cells to also mediate target-antigen dependent hematotoxicity, however, in contrast to conventional CAR T constructs, the AdCAR platform allows limited exposure and thereby a possibly beneficial safety profile.

The AdCAR T-cell technology relies on AMs of low molecular weight (Fab molecules), which, in contrast to Ab-based AM formats, have substantially shorter half-lives (1–2 h vs days to weeks) [32, 33]. Interestingly, a recent report highlighted the possibility for preventing or arresting CRS in an adenocarcinoma mouse model using low-molecular-weight AMs [34]. Consistent with that, we readily observed cessation of AdCAR T-cell activation in pAML co-culture assays when αCD33-AM_Fab_ addition was stopped, suggesting similar efficacy of our platform in controlling therapy-related toxicity. Future studies will need to address, if intermittent application of AdCAR T cells will allow restoration of healthy hematopoiesis within the treatment free intervals.

In addition to stability and renal clearance of the AM [35], we identified receptor-mediated endocytosis as another variable that determines bioactivity and serum half-lives. As the majority of AML-associated target antigens are myeloid lineage antigens, the binding of which leads to internalization, we studied this mechanism as a potential antigen sink that affects AM pharmacokinetics. As hypothesized, receptor-mediated endocytosis led to AM depletion, thereby influencing AdCAR T-cell potency. While on a systemic level the elimination is likely governed by renal clearence of these small molecular weight AMs, we think that AM internalization on a cellular level contributes to the controlled activation/termination of AdCAR T cells. We believe this to be a broadly applicable mechanism in T-cell-based immunotherapy of myeloid malignancies, that potentially applies to BsAbs as well as AdCAR T cells. In fact, antigen sink effects might have contributed to the need for much higher doses of the CD33-targeting BsAb AMG330 compared to blinatumomab in a recent phase I trial [24]. Future AM dosing regimens should therefore take this effect into account.

Another emerging factor for the failure of T-cell-based immunotherapies is T-cell dysfunction due to chronic antigen exposure [15, 16] or tonic CAR T-cell signaling [18, 19]. In a study by Weber et al., [19], transient rest was shown to reinvigorate exhausted, tonically signaling GD2-CAR T cells through epigenetic remodeling. Importantly, we recently identified similar features for BsAbs [22], indicating that T-cell dysfunction can be induced through different signaling pathways. Based on these observations, we hypothesized that AdCAR T cells follow the same principles. However, the short half-lives of the AMs provide the AdCAR T cells an on–off switch, an inherent advantage over conventional CAR T cells that makes implementation of resting periods through TFIs possible (comparable to intermittent BsAb treatment).

Indeed, in an AML-optimized long-term culture system recapitulating 21 days of continuous AdCAR T-cell stimulation, we observed progressive loss of effector functions. Interruption of AM exposure for 7 days resulted in rejuvenation of AdCAR T cells compared to CONT-stimulated cells, which was reflected by restored effector function and transcriptional remodeling.

When we compared AdCAR T cells after intermittent AM exposure to BsAb-activated T cells, also after a TFI [22], we observed differences in the transcriptome. Although puzzling at first, we believe that the two scenarios cannot be compared as AdCAR T cells are stimulated during the initial production protocol. Interestingly, more recent developments in CAR T-cell production have focused on maintaining a more naïve-like phenotype, thereby conserving the original T-cell subset composition at the time of leukapheresis. In that sense, our AdCAR T cells rather resemble conventional CAR T cells, suggesting that optimized manufacturing protocols will likely influence the efficacy of AdCAR T-cell rejuvenation by TFIs [8, 36, 37].

In summary, we established a highly potent and flexible AdCAR T-cell platform for T-cell-based immunotherapy in AML. Owing to its modular design and the use of low-molecular-weight adapters, the platform allows targeting of different AML-associated target antigens and controlled T-cell activation. The possibility of intermittent activation counteracts AdCAR T-cell exhaustion. Our data support the use of AdCAR T cells in an early clinical trial for patients with relapsed/refractory AML, and addresses questions that are most likely pertinent to other disease entities.

## Supplementary information

Supplementary material

Supplementary Figure 1

Supplementary Figure 2

Supplementary Figure 3

Supplementary Figure 4

Supplementary Figure 5

## Data Availability

The RNA-seq data discussed in this publication have been deposited in the GEO database under the accession code GSE221070. The datasets generated and/or analyzed during this study are available from the corresponding author upon reasonable request.
