# Supplementary material for: Adapter CAR T cells to counteract T-cell exhaustion and enable flexible targeting in AML
Source: Leukemia. 2023 Apr 27;37(6):1298–310. doi: 10.1038/s41375-023-01905-0 (PMC10244166; doi:10.1038/s41375-023-01905-0)
Supplement: Supplementary file 1 — Supplementary material [file 41375_2023_1905_MOESM1_ESM.docx]

**Supplementary Material & Methods**

**AdCAR construction**

Lentiviral transfer plasmids were generated by cloning the anti-LLE scFv (hBio3 or mBio2) onto a CAR sequence comprising of a hIgG4 stalk, a hCD8a transmembrane domain, CD28_4-1BB (mBio2) or 4-1BB only (hBio3) as costimulatory domain and hCD3ζ. The conventional CD33CAR plasmid was kindly provided by Dina Schneider (Lentigen, A Miltenyi Biotec Company, Gaithersburg, MD, United States). Lentivirus (LV) was produced in HEK293T cells after transfection with polyethylenimine. Virus was pelleted form the supernatant by centrifugation (4500g, 24 h) and resuspended with medium before storage at -80°C. LV titers were determined with SupT1 cells in a flow-cytometry-based assay using the respective CAR detection reagent.

**Adapter molecule generation**

Fab molecules were expressed in Chinese Hamster Ovary (CHO) or Pichia pastoris expression hosts and purified by affinity chromatography (Ni-NTA Agarose or CaptureSelect™ CH1-XL Affinity Matrix, ThermoFisher). Purified Fabs were reduced for 1 h at 21°C with DTT (10 mM) in PBS Buffer (pH 7.8) containing EDTA (5 mM). Excess DTT was removed by gel filtration (Sephadex G25) and reduced Fabs were reacted with 1.8 molar equivalents ETAC biotinylation reagent (Miltenyi Biotec) for 2 h at 21°C. Excess reagent was removed by gel filtration (Sephadex G25). Adapter molecules were sterile filtered (0,22 µm Millex-GV, Merck Millipore) and stored in aliquots at -80°C. Degree of biotinylation was determined by reducing SDS-PAGE and intact mass determination was performed using LC/MS (microTOF QII, Bruker).

**Patients**

Peripheral blood (PB) or bone marrow (BM) samples were collected from healthy donors (HDs) and AML patients at initial-diagnosis (ID) after obtaining written informed consent, in accordance with the Declaration of Helsinki and with the approval of the Institutional Review Board of the Ludwig Maximilian University (Munich, Germany). Patient characteristics are summarized in Table 1.

**AdCAR T-cell manufacture**

Mononuclear cells from PB or BM of HDs or patients with pAML were isolated by density gradient centrifugation. CD8^+^ and CD4^+^ T cells were isolated sequentially according to Miltenyi Biotec’s CD8/CD4 Micro Bead protocols and cultured in TexMACS medium (Miltenyi Biotec) supplemented with 10 ng/ml IL-7/IL-15 (Peprotech, Hamburg, Germany). T cells were mixed at a CD8^+^/CD4^+^ ratio of 1:1 and activated for 24 h with human TransAct (1:100). Lentiviral transduction was performed at a multiplicity of infection of 5–10 and cells were cultured for 12–14 days until cryo-preservation. Transduction efficiency was assessed by biotin-PE or LNGFR-PE/FITC (Miltenyi Biotec) staining and subsequent flow-cytometric analysis. In cases involving pAML and HD-BM-derived AdCAR T cells, the cells were negatively isolated using an EasySep Human T-Cell Isolation Kit (Stemcell, Vancouver, Canada) according to the manufacturer’s instructions. Comparable transduction efficiencies were observed throughout all AdCAR experiments.

**Cell cultivation**

AML cell line cultivation, co-culturing and functional assays were performed in R10 culture medium. Primary AML samples were cultured in complete blast medium. Human T cells for generating AdCAR T cells were cultured in CAR T-cell medium. All cells were cultured at 37 °C, 5% CO_2_, and regularly tested for mycoplasma. Cell lines were purchased from the ATCC and cell line authentication was performed regularly. Lists of media and cell lines are provided in Tables S1 and S2, respectively.

**Immunophenotyping and quantification of cytokines**

*Surface marker staining*

Immunophenotyping was performed by using flow-cytometry. The cells were washed with FACS buffer and stained for 20 min with either the LIVE/DEAD Fixable Aqua Dead Cell Stain Kit (Invitrogen, Vienna, Austria) or 7AAD and the respective antibody (Table S3). Samples were analyzed on Cytoflex S or Cytoflex LX instruments (Beckman Coulter). Median fluorescence intensity (MFI) values were determined using FlowJo (version 9.6; Tree Star Inc., Ashland, OR) and the MFI ratios were calculated based on the corresponding isotype controls.

*Cytokine quantification*

Cytokine secretion into the supernatant of co-cultures was determined on cytometric bead arrays (CBAs) using a Th1/Th2 Cytokine Kit II (BD Biosciences, Franklin Lakes, NJ) according to the manufacturer’s instructions.

Intracellular granzyme B staining was performed by treating the cells for 4 h with 10 ng/ml brefeldin A and 25 nM monensin (all Sigma–Aldrich, St. Louis, MO) and subsequently staining with LIVE/DEAD Fixable Aqua Dead Cell Stain Kit and antibodies against surface markers. For intracellular staining, the cells were permeabilized using a BD Cytofix/Cytoperm Kit (BD Biosciences).

**Confocal microscopy**

Images were acquired on a Nikon TiE microscope equipped with a Yokogawa CSU-W1 spinning-disk confocal unit (50 µm pinhole size), an Andor Borealis illumination unit, an Andor ALC600 laser beam combiner (405 nm/488 nm/561 nm/640 nm), and an Andor IXON 888 Ultra EMCCD camera. The microscope was controlled by software from Nikon (NIS Elements, ver. 5.02.00). Cells were imaged in an environmental chamber maintained at 37°C with 5% CO_2_ (Oko Labs), using a Nikon PlanApo 100x/1.5 NA oil immersion objective and a Perfect Focus System (Nikon). Image stacks were recorded with a step size of 1.0 µm and a pixel size of 131 nm. Hoechst 33342 and pHrod red avidin were excited using the 405 and 561 nm laser lines, respectively. Fiji software (ImageJ 1.51j) was used to analyze images.

**CRISPR–Cas9-based knockout generation**

The CD33-knockout MV4-11 cell line was generated by CRISPR–Cas9 with electroporation using the Lonza 4D Nucleofector X Unit (Lonza, Basel, Switzerland). RNP assembly was performed by annealing synthetic crRNA:tracrRNA (IDT, Coralville, IA) at 95°C for 5 min followed by incubation at 22°C for 30 min. CD33 crRNA was designed according to Ref. [1]. One hundred picomoles of assembled RNA was mixed with 40 pmol Alt-R S.p. Cas9 nuclease V3 (IDT) for 10 min at RT. For nucleofection, 300,000 cells were resuspended in 20 µl of supplemented nucleofection buffer using the SF Cell Line 4D-Nucleofector X Kit S (program DJ-100). Cells were transferred to 48-well plates and recovered in serum-free RPMI (without supplements) for 30 min at 37°C and supplemented with complete R10. The cells were stained for CD33 expression and the CD33-negative population was sorted twice using a MoFlo Astrios EQ cell sorter (Beckman Coulter).

**Influence of repeated AM internalization on target antigen expression levels**

AML cell lines were incubated for 15 min at 4°C with αCD33-AM_Fab_. Unbound AM was removed, cells were warmed to 37°C and stained at timepoints *t* = 0, 24 and 72 h with anti-biotin-PE to detect surface-bound AM. In addition, assessment of CD33 target antigen levels and live–dead discrimination were performed as previously described. After 72 h, the experimental procedure was repeated for another two cycles.

***In vivo* studies**

For *in vivo* experiments, 7- to 9-week-old female NOD.Cg-*Prkdc^scid^* *Il2rg^tm1Wjl^*/SzJ (NSG) mice from Charles River Laboratories (Bar Harbor, ME) were used and kept in individually ventilated cages. Mice were engrafted with 7 × 10^4^ OCI-AML-2 cells stably expressing a firefly luciferase gene, via tail vein injection on day −5. Tumor cell engraftment was monitored by *in vivo* bioluminescence imaging (BLI) on day −1, and animals were randomized according to tumor burden. For BLI, mice were intraperitoneally (i.p.) injected with 3 mg of D-luciferin (ZellBio GmbH, Lonsee, Germany), and a 6 min uptake period followed. BLI scans were acquired on an IVIS Spectrum Optical Imaging system (Perkin Elmer) for a maximum of 60 s, and total flux (photons per second) was determined. Regions of interest (ROI) were defined around the whole animal using Living Image Software 4.5.2 (Perkin Elmer).

Six million AdCAR T cells (mBio2) or conventional CD33CAR T cells [2] were injected intravenously on day 0. Cell numbers were adjusted to match identical transduction efficiencies. The AM αCD33-AM_Fab_ (100 µg) was administered daily by i.p. injection. Tumor growth was monitored twice per week by BLI. All mice were sacrificed when the control mice reached end-point criteria in accordance with the approval granted by the local authorities (written approval: 81-02.04.2018.A096).

**Bulk RNA sequencing**

Long-term AdCAR T-cell stimulation and TFI experiments were set up as described in the main text. After isolation of T cells, the AdCAR-positive cells were sorted. Five thousand AdCAR T cells were transferred to 50 µl of Buffer RLT Plus (Qiagen, Venlo, Netherlands) containing 1% β-mercaptoethanol and stored at −80°C. RNA isolation and library preparation were performed according to the Prime-seq protocol [3]. A step-by step protocol can be found on protocols.io (<https://doi.org/10.17504/protocols.io.s9veh66>).

**Data visualization and statistical analysis**

Data was visualized using Prism 9 version 9.2.0 (283) (GraphPad Software Inc.), Adobe Illustrator version 25.2.3 (Adobe Inc.), and BioRender.com. Confocal images were analyzed with Fiji (ImageJ 1.51j). Statistical analyses were performed using Prism 9.

**Supplementary tables**

**Supplementary Table 1: Media and buffers**

| **Description** | **Ingredients** |
| --- | --- |
| 2x complete blast medium | 2x blast medium with 40 ng/ml rh IL-3, rh TPO, rh G-CSF, 114.4 µM β-mercaptoethanol |
| Blast medium | Alpha MEM (PAN Biotech) with 12.5% FBS, 12.5% horse serum, 1% pen–strep–L-glutamine |
| CAR T-cell medium | Human TexMACS (Miltenyi Biotec) + 1% pen–strep–L-glutamine and 10 ng/ml IL-7/IL-15 (Peprotech) |
| Complete blast medium | Blast medium with 20 ng/ml rh IL-3, rh TPO, rh G-CSF, 57.2 µM β-mercaptoethanol |
| Cryo-conservation medium | Cell lines: RPMI 1640 + 10% DMSO + 10% FCS  Primary AML: RPMI 1640 + 10% DMSO + 45% FCS |
| FACS buffer | PBS + 0.1% BSA and 2 mM EDTA |
| FACS Fix | 47.3 ml FACS buffer + 2.7 ml formaldehyde |
| HD blast medium | Blast medium with 20 ng/ml rh IL-3, rh IL-6, rh TPO, rh GM-CSF, FLT3L, SCF, 57.2 µM β-mercaptoethanol |
| R10 | RPMI 1640 (PAN Biotech) + 10% FCS, 1% pen–strep–L-glutamine, 1% HEPES |

**Supplementary Table 2: Cell lines**

| **Name** | **Species** | **Cell type** |
| --- | --- | --- |
| HL-60 | Human | AML |
| MS5 | Mouse | Stromal cells |
| MV4-11 | Human | AML |
| NALM-6 | Human | B-cell precursor leukemia |
| OCI-AML-2 | Human | AML |
| OCI-AML-3 | Human | AML |

**Supplementary Table 3: Antibodies for flow-cytometry**

| **Antibody** | **Clone** | **Conjugate** | **Manufacturer** | **Cat. #** |
| --- | --- | --- | --- | --- |
| CD2 | RPA-2.10 | PerCP/Cy5.5 | BioLegend | 300216 |
| CD2 | REA1130 | APC | Miltenyi Biotec | 130-119-509 |
| CD2 | TS1/8 | BV421 | BioLegend | 309218 |
| CD2 | RPA-2.10 | FITC | BioLegend | 300206 |
| CD3 | HIT3a | PerCP/Cy5.5 | BioLegend | 300328 |
| CD4 | RPA-T4 | PC7 | BioLegend | 300512 |
| CD4 | OKT4 | FITC | BioLegend | 317408 |
| CD4 | REA623 | VioGreen | Miltenyi Biotec | 130-113-230 |
| CD8 | REA734 | APC-Vio770 | Miltenyi Biotec | 130-110-681 |
| CD8 | SK1 | APC-Cy7 | BioLegend | 344714 |
| CD45RA | REA1047 | VioBlue | Miltenyi Biotec | 130-117-854 |
| CCR7 | G043H7 | PE | BioLegend | 353204 |
| CD45 | 2D1 | FITC | BioLegend | 368508 |
| CD45 | RREA747 | VioBlue | Miltenyi Biotec | 130-110-637 |
| CD33 | WM-53 | PC7 | Invitrogen | 25-0338-42 |
| CD33 | REA775 | APC | Miltenyi Biotetc | 130-111-020 |
| CD33 | REA775 | PE | Miltenyi Biotec | 130-111-019 |
| CD123 | REA918 | APC | Miltenyi Biotec | 130-115-358 |
| CLL-1 | REA431 | PE-Vio770 | Miltenyi Biotec | 130-106-436 |
| PD1 | EH12.2H7 | FITC | BioLegend | 329904 |
| PD1 | EH12.2H7 | PC7 | BioLegend | 329918 |
| LAG-3 | REA351 | APC | Miltenyi Biotec | 130-119-567 |
| TIM3 | F38-2E2 | BV421 | BioLegend | 345008 |
| Granzyme B | QA16A02 | PE | BioLegend | 372208 |
| CD271 (LNGFR) | REA844 | PE | Miltenyi Biotec | 130-112-790 |
| Anti-biotin | REA746 | PE | Miltenyi Biotec | 130-110-951 |

**Supplementary Table 4: Reagents**

| **Name** | **Manufacturer** | **Cat. #** |
| --- | --- | --- |
| 7-AAD Staining Solution | Miltenyi Biotec | 130-111-568 |
| Alpha-MEM | PanBiotech | P04-21500 |
| Alt-R S.p. Cas9 Nuclease V3 | IDT | 1081058 |
| Anti-mouse IgG,k CompBeads | BD | 51.90.9001229 |
| Anti-REA CompBeads | Miltenyi Biotech | 130-104-693 |
| Biotin-PE | Miltenyi Biotec | Non-commercial |
| Brefeldin A | Sigma–Aldrich | B7651-5MG |
| Buffer RLT Plus | Qiagen | 1053393 |
| CD33 CAR Detection Reagent | Miltenyi Biotec | 130-127-642 |
| D-Luciferin, potassium Salt | ZellBio GmbH | PubChem Chemical ID: 44134804 |
| DMSO | Serva | 20385.01 |
| DPBS | PanBiotech | P04-36500 |
| FcR Blocking Reagent | Miltenyi Biotec | 130-059-901 |
| Fetal bovine serum | Thermo Fisher Scientific | 10270106 |
| Ficoll Histopaque-1077 Hybri-Max | Sigma–Aldrich | H8889-500ML |
| Formaldehyde solution | Roth | 7398.1 |
| HEPES buffer solution (1 M) | Gibco Life Technologies | 15630-056 |
| Hoechst 33342 | Enzoo Life Sciences | ENZ-52401 |
| Horse serum | Sigma–Aldrich | H1270 |
| Human TexMACS | Miltenyi Biotec | 130-097-196 |
| MACS BSA stock solution | Miltenyi Biotec | 130-091-376 |
| Monensin, sodium salt | Sigma–Aldrich | M5273-1G |
| Penicillin–streptomycin–L-glutamine, 100x | Thermo Fisher Scientific | 10378016 |
| pHrodo Red Avidin | Thermo Fisher Scientific | P35362 |
| rh FLT3L | Peprotech | 300-19 |
| rh G-CSF | Peprotech | 300-23 |
| rh GM-CSF | Peprotech | 300-03 |
| rh IL-15 | Peprotech | 200-15 |
| rh IL-3 | Peprotech | 200-3 |
| rh IL-7 | Peprotech | 200-7 |
| rh SCF | Peprotech | 300-007 |
| rh TPO | Peprotech | 300-18 |
| RPMI 1640 | PanBiotech | P04-16500 |
| Sodium heparin | Ratiopharm | N68542.05-Z01 |
| Sodium pyruvate (100 mM), 100X | Gibco Life Technologies | 11360-70 |
| Trypan blue | Thermo Fisher Scientific | T10282 |
| UltraPure 0.5 M EDTA | Invitrogen | 15575-038 |
| β-Mercaptoethanol | Sigma–Aldrich | M6250 |
| µ-Slide 2-well, glass bottom | Ibidi | 80287 |

**Supplementary Table 5: Kits**

| **Name** | **Manufacturer** | **Cat. #** |
| --- | --- | --- |
| CD4 MicroBeads, human | Miltenyi Biotec | 130-045-101 |
| CD8 MicroBeads, human | Miltenyi Biotec | 130-045-201 |
| Cytofix/Cytoperm Fixation/Permeablization Kit | BD Biosciences | 554714 |
| EasySep Human CD3 Positive Selection Kit II | Stemcell Technologies | 17851 |
| EasySep Human T Cell Isolation Kit (Stemcell) | Stemcell Technologies | 17951 |
| Human Th1/Th2 Cytokine Kit II | BD Biosciences | 551809 |
| LIVE/DEAD Fixable Aqua Dead Cell Stain Kit | Invitrogen | L34957 |
| SF Cell Line 4D-NucleofectorTM X Kit S | Lonza | V4XC-2032 |

**Supplementary Figure Legends**

**Figure S1: AdCAR T-cell characterization and functional validation**

**A)** MFI ratio of CD33, CD123, and CLL-1 expressed on pAML cells assessed by surface marker staining with biotinylated AMs and subsequent secondary staining (*n* = 32). MFI ratios were calculated based on corresponding controls without AMs. **B)** T-cell subsets measured by CD45RA and CCR7 co-expression, before (left) and after (right) T-cell activation, transduction and expansion for up to 14 days (*n* = 6–9). **C)** Representative gating strategy used to assess AdCAR T-cell-mediated cytotoxicity. **D)** AdCAR T-cell-mediated cytotoxicity after 48 h (*n* = 3–5) against the AML cell lines MV4-11, HL-60, and OCI-AML-3 co-cultured with 10 ng/ml αCD33-AM_Fab_ at varying E:T ratios. Specific lysis was calculated relative to the mock T-cell condition. **E)** Cytotoxicity of AMs at 1 µg/ml assessed after 48 h in co-cultures with mock T cells and respective target-antigen-positive cell lines (E:T = 1:1). AdCAR T cells in combination with 10 ng/ml AM served as positive controls (*n* = 3–5). **F)** AdCAR T-cell-mediated cytotoxicity after 48 h (*n* = 6–10) against the AML cell lines MV4-11, HL-60, and OCI-AML-3 (E:T = 1:1) was compared for Fab- versus Ab-based AM formats at concentrations ranging from 1 pg/ml to 1000 ng/ml. Specific lysis was calculated relative to the mock T-cell condition. Data are plotted as mean ± SEM. Statistical analysis: paired *t*-test; ns *p* > 0.05; * *p* < 0.05; ** *p* < 0.01; *** *p* < 0.001; **** *p* < 0.0001.

**Figure S2: AdCAR-mediated cytotoxicity against pAML cells: impact of receptor-mediated internalization on AdCAR T-cell efficacy**

**A)** T-cell proliferation was assessed by flow-cytometry during 12 days co-culture of AdCAR T cells and pAML cells in the presence of 100, 10 or 1 ng/ml αCD33-AM_Fab_. The fold change of CD2^+^ cells at the indicated time points relative to day 0 (*n* = 3) is shown. **B)** Corresponding TIM3 expression as MFI ratio, as well as the percentage of PD1, LAG-3, TIM3 triple-positive T cells (*n* = 2–3) after a single stimulation of AdCAR T cells with 100 ng/ml αCD33-AM_Fab_. **C)** Comparison of cytotoxicity and T-cell proliferation after 72 h (*n* = 3) of AdCAR T cells generated from HD T cells versus pAML-derived T cells in co-culture with OCI-AML-3 cells (E:T = 1:1) in the presence of 10 ng/ml αCD33-AM_Fab_. **D)** Flow-cytometry study of internalization of 500 ng/ml αCD33-AM_Fab_ coupled to pHrodo Red Avidin for 4 h at 37°C on MV4-11 cells. Wildtype MV4-11 cells were compared to CD33-deficient and wildtype MV4-11 cells treated with 25 nM monensin. Monensin was added 30 min before the addition of the AM (*n* = 3). Uncoupled pHrodo Red Avidin served as a negative control. MFI ratios were calculated relative to control conditions at 4°C. **E)** Quantitative representation of indirect AM internalization on pAML cells (*n* = 5). **F)** Influence of three repetitive αCD33-AM_Fab_ (100 ng/ml) cycles of internalization on the level of CD33 receptor in MV4-11 and OCI-AML-3 cells (*n* = 3). CD33 levels are indicated as MFI ratio (black curve). MFI ratios for surface-bound αCD33-AM_Fab_ are plotted in red. Data are plotted as mean ± SEM. Statistical analysis: paired *t*-test; ns *p* > 0.05; * *p* < 0.05; ** *p* < 0.01; *** *p* < 0.001; **** *p* < 0.0001.

**Figure S3: AdCAR T cells allow for sequential targeting of pAML cells**

**A,B)** T-cell proliferation and T-cell activation on day 9 of co-culture (corresponding to data in Fig. 3A,B). Proliferation is represented by fold change in CD2^+^ cells compared to conditions without AM. T-cell activation is represented as the percentage of PD1, LAG-3, TIM3 triple-positive T cells. **C,D)** Long-term co-cultures of AdCAR T cells and pAML cells (E:T = 1:10) for 12 days. pAML cell counts over time are plotted as normalized target cell counts relative to starting conditions on day 0 (*n* = 4–7). αCD19-AM_Fab_ was replenished every third day at 10 ng/ml and served as a control. αCLL-1-AM_Fab_ was either applied once (10 ng/ml) or every third day until day 6. On days 6 and 9, the AM dose was either kept at 10 ng/ml or increased to 100 ng/ml. Alternatively, AMs were switched to those with different target specificity (αCD33-AM_Fab_ or αCD123-AM_Fab_) on days 6 and 9 (10 or 100 ng/ml). **E)** Corresponding AdCAR T-cell-mediated cytotoxicity on day 12 of co-culture. Specific lysis was calculated relative to AdCAR T-cell condition in the absence of AMs. **F/G)** Corresponding T-cell proliferation and T-cell activation on day 9 of co-culture. Proliferation is represented by fold change in CD2^+^ cells compared to conditions in the absence of AM. T-cell activation is represented as the percentage of PD1, LAG-3, TIM3 triple-positive T cells. Data are presented as mean ± SEM. Statistical analysis: Ordinary one-way ANOVA with Dunnett’s comparison; ns *p* > 0.05; * *p* < 0.05; ** *p* < 0.01; *** *p* < 0.001; **** *p* < 0.0001.

**Figure S4: Treatment-free intervals prolong AdCAR T-cell function *in vitro***

**A)** Percentage of CD8^+^ T cells expressing PD1 or LAG-3 on day 17 of co-culture (*n* = 12). **B)** AdCAR transgene expression on CD8^+^ T cells over time, assessed by biotin-PE staining (*n* = 12). **C)** Development of continuously stimulated CD8^+^ T-cell subset composition, assessed by CD45RA and CCR7 staining during 21 days of co-culture with OCI-AML-3 (*n* = 12). Subset compositions of continuously versus intermittently stimulated AdCAR T cells are shown separately for days 14 and 21. Statistical analysis: paired *t*-test; ns *p* > 0.05; * *p* < 0.05; ** *p* < 0.01; *** *p* < 0.001; **** *p* < 0.0001.

**Figure S5: Treatment-free intervals lead to transcriptional reprograming of AdCAR T cells**

**A)** Volcano plot of differentially expressed genes (DEGs) in day 21 TFI-treated versus CONT-treated AdCAR T cells; *p* < 0.01. Selected genes are highlighted in blue (downregulated) or red (upregulated). **B)** Heatmap with hierarchical clustering of the top 100 DEGs in day 21 TFI-treated versus CONT-treated AdCAR T cells; *p* < 0.01. Selected genes are indicated. **C)** Hallmark gene set analysis of day 14 versus day 21 TFI-treated AdCAR T cells; *p* < 0.05. NES = normalized enrichment score. **D)** Gene set enrichment analysis (GSEA) of day 21 TFI-treated versus CONT-treated AdCAR T cells using MSigDB and the gene set GSE9650_EFFECTOR_VS_MEMORY_CD8_TCELL_UP.

**Supplementary references**

1. Kim MY, Yu KR, Kenderian SS, Ruella M, Chen S, Shin TH, et al. Genetic Inactivation of CD33 in Hematopoietic Stem Cells to Enable CAR T Cell Immunotherapy for Acute Myeloid Leukemia. Cell. 2018;173(6):1439-53 e19.

2. Schneider D, Xiong Y, Hu P, Wu D, Chen W, Ying T, et al. A Unique Human Immunoglobulin Heavy Chain Variable Domain-Only CD33 CAR for the Treatment of Acute Myeloid Leukemia. Front Oncol. 2018;8:539.

3. Janjic A, Wange LE, Bagnoli JW, Geuder J, Nguyen P, Richter D, et al. Prime-seq, efficient and powerful bulk RNA sequencing. Genome Biology. 2022;23(1):88.
