# Supplementary material for: Adapter CAR T cells to counteract T-cell exhaustion and enable flexible targeting in AML
Source: Leukemia. 2023 Apr 27;37(6):1298–310. doi: 10.1038/s41375-023-01905-0 (PMC10244166; doi:10.1038/s41375-023-01905-0)
Supplement: Supplementary file 2 — Supplementary Figure 1 [file 41375_2023_1905_MOESM2_ESM.pdf]

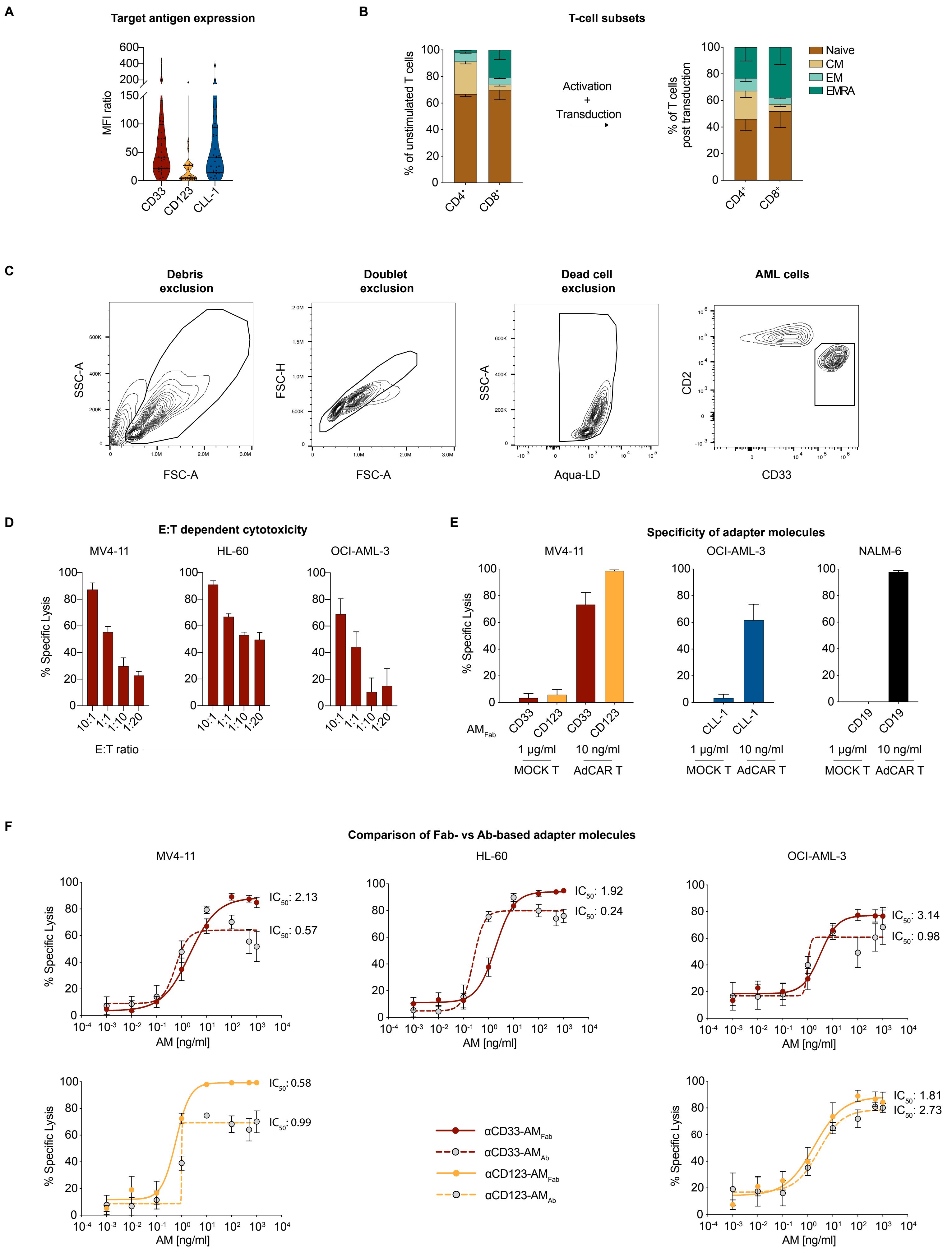
A
Target antigen expression
MFI ratio
CD33
CD123
CLL-1
B
T-cell subsets
% of unstimulated T cells
Activation
+
Transduction
% of T cells
post transduction
Naive
CM
EM
EMRA
CD4+
CD8+
C
Debris
exclusion
Doublet
exclusion
Dead cell
exclusion
AML cells
SSC-A
FSC-A
FSC-H
FSC-A
SSC-A
Aqua-LD
CD2
CD33
D
E:T dependent cytotoxicity
MV4-11
HL-60
OCI-AML-3
% Specific Lysis
10:1
1:1
1:10
1:20
E:T ratio
E
Specificity of adapter molecules
MV4-11
OCI-AML-3
NALM-6
% Specific Lysis
AMFab
CD33
CD123
CD33
CD123
CLL-1
CLL-1
CD19
CD19
1 µg/ml
10 ng/ml
MOCK T
AdCAR T
F
Comparison of Fab- vs Ab-based adapter molecules
MV4-11
HL-60
OCI-AML-3
% Specific Lysis
AM [ng/ml]
IC50: 2.13
IC50: 0.57
IC50: 1.92
IC50: 0.24
IC50: 3.14
IC50: 0.98
IC50: 0.58
IC50: 0.99
IC50: 1.81
IC50: 2.73
αCD33-AMFab
αCD33-AMAb
αCD123-AMFab
αCD123-AMAb
