# Supplementary figures and images for: Adapter CAR T cells to counteract T-cell exhaustion and enable flexible targeting in AML
Source: Leukemia. 2023 Apr 27;37(6):1298–310. doi: 10.1038/s41375-023-01905-0 (PMC10244166; doi:10.1038/s41375-023-01905-0)

**A**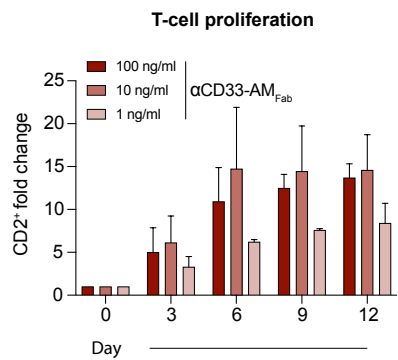**B**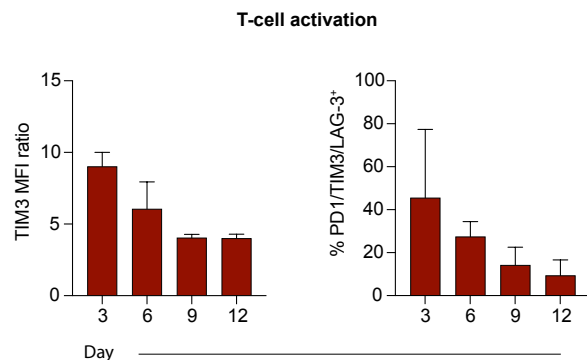**C**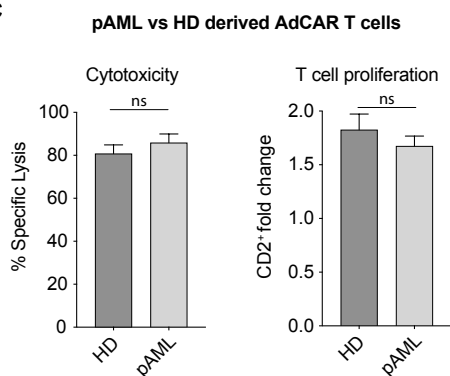**D**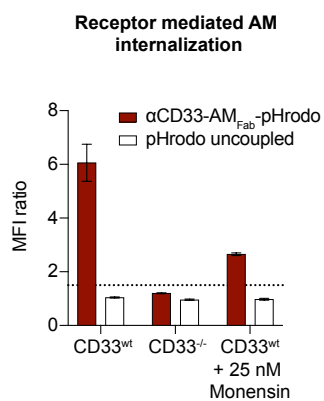**E**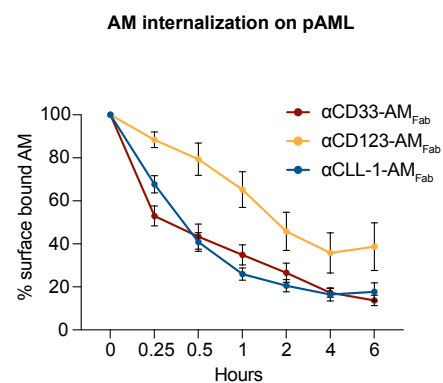**F**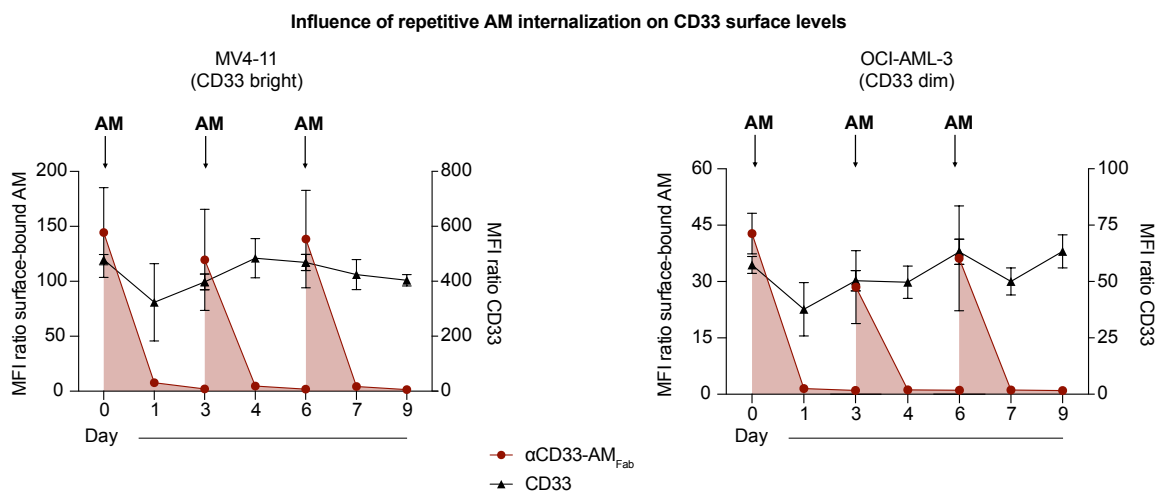

Supplement: Supplementary file 3 — Supplementary Figure 2 [file 41375_2023_1905_MOESM3_ESM.pdf]

A

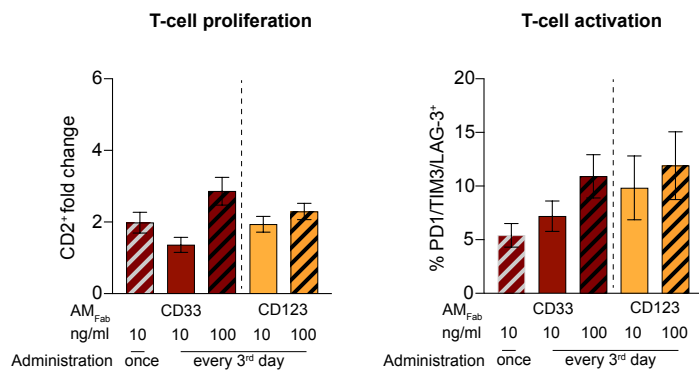

B

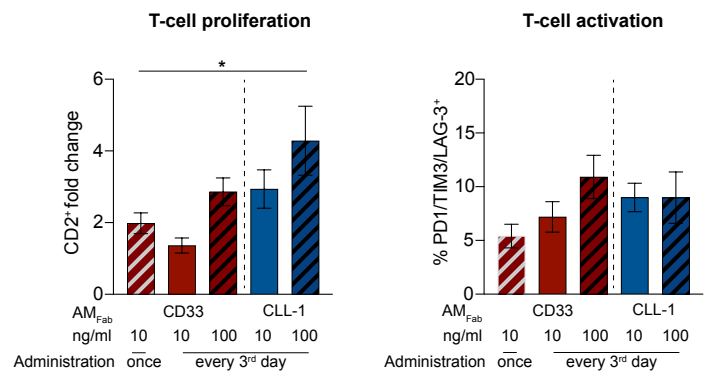

C

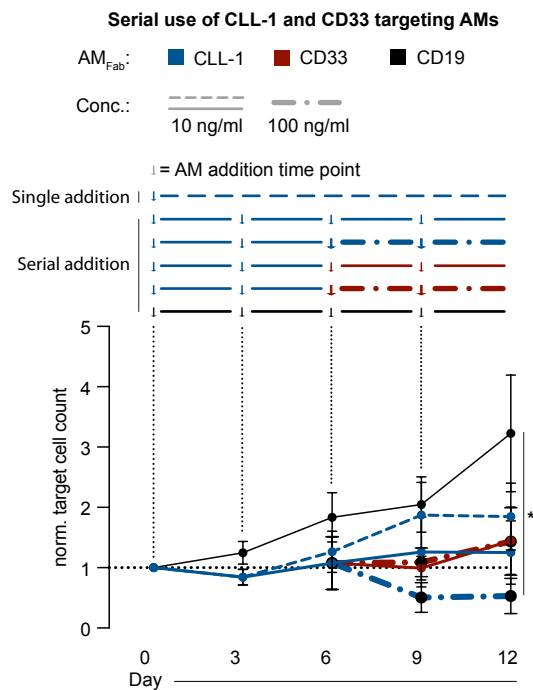

D

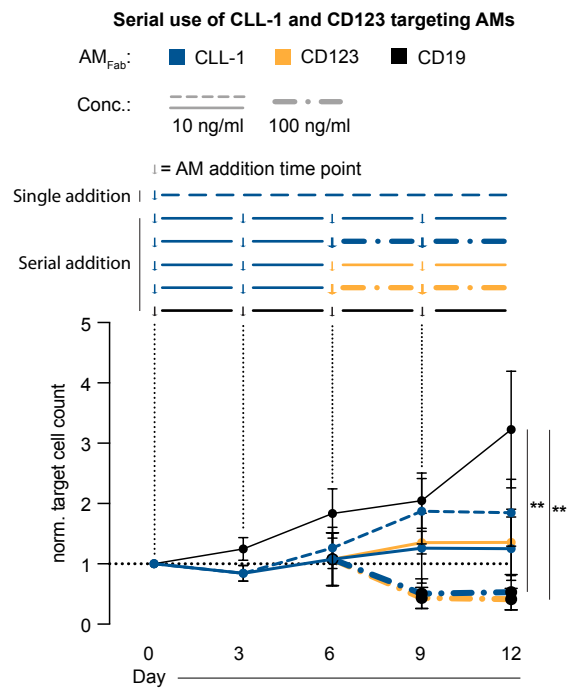

E

### Cytotoxicity on day 12 of pAML co-culture

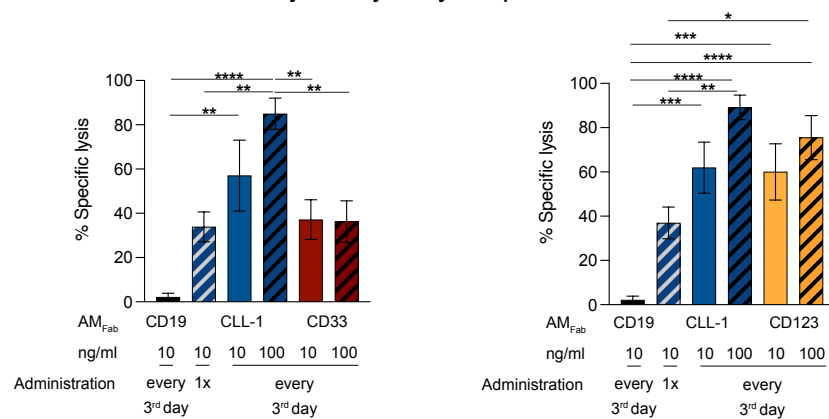

F

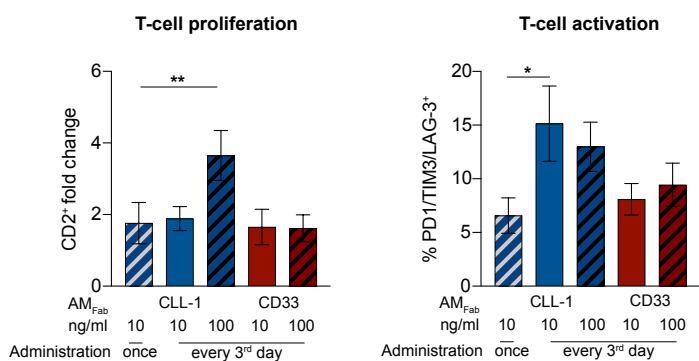

G

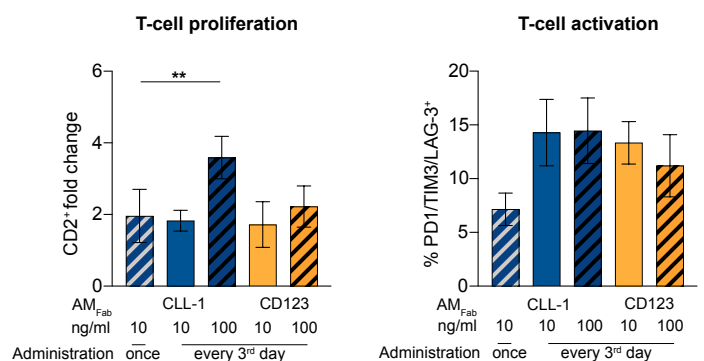

Supplement: Supplementary file 4 — Supplementary Figure 3 [file 41375_2023_1905_MOESM4_ESM.pdf]

**A**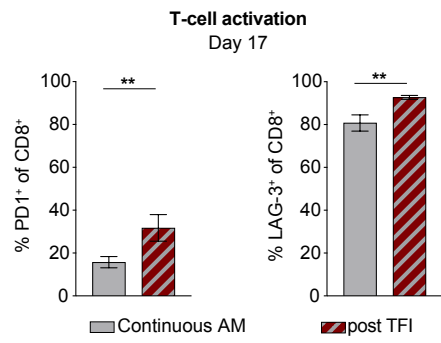**B**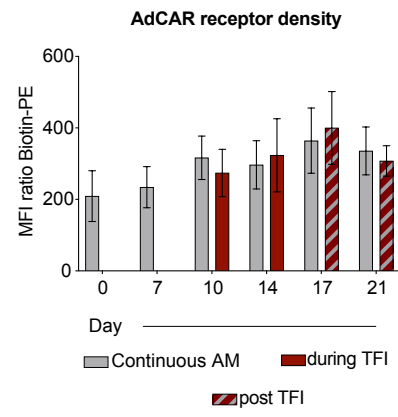**C**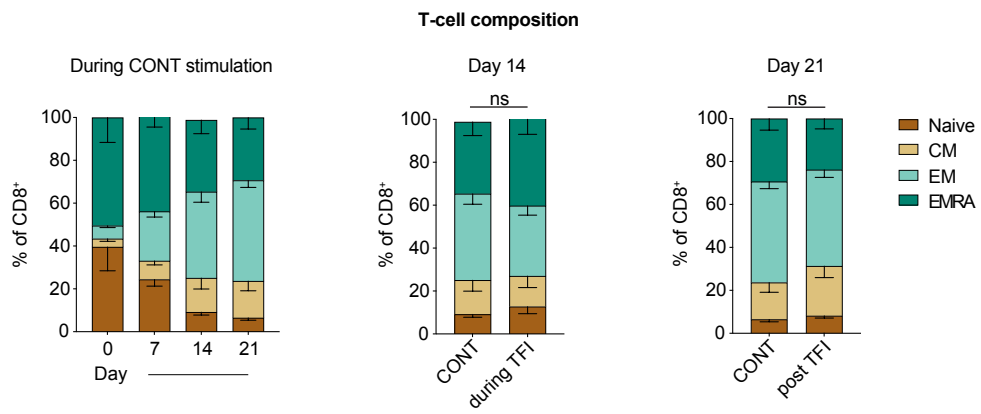

Supplement: Supplementary file 5 — Supplementary Figure 4 [file 41375_2023_1905_MOESM5_ESM.pdf]

A

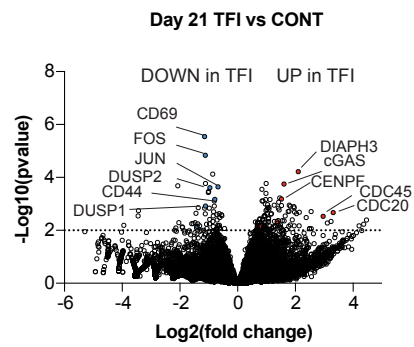

B

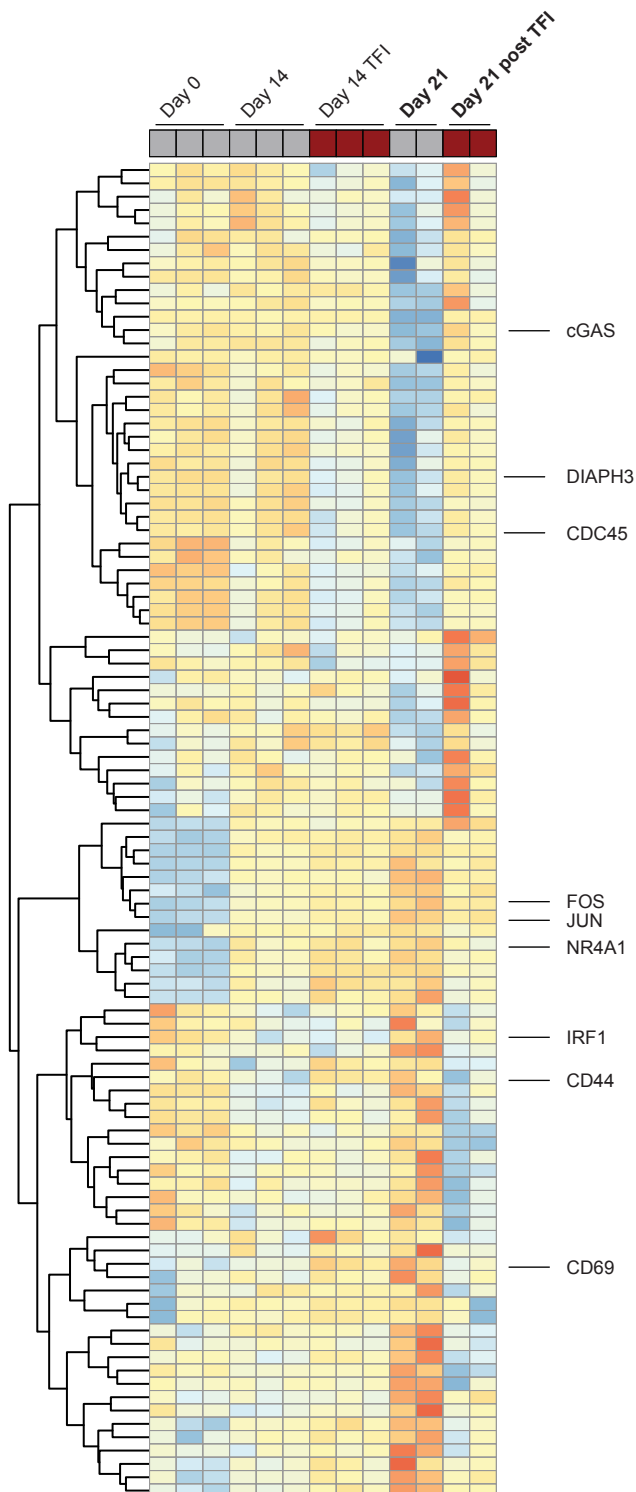

C

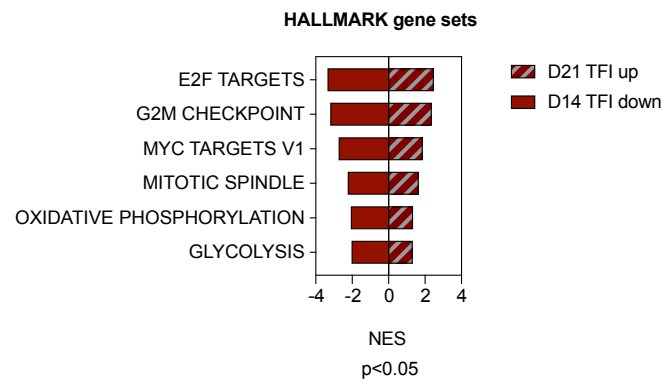

D

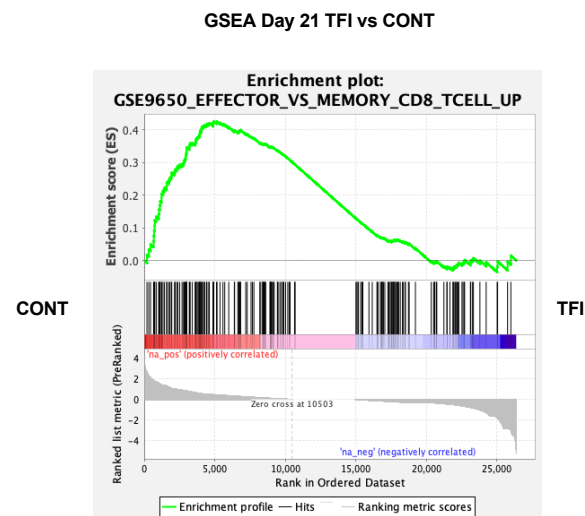

Supplement: Supplementary file 6 — Supplementary Figure 5 [file 41375_2023_1905_MOESM6_ESM.pdf]
